# Reversible unfolding of infectious prion assemblies reveals the existence of an oligomeric elementary brick

**DOI:** 10.1371/journal.ppat.1006557

**Published:** 2017-09-07

**Authors:** Angélique Igel-Egalon, Mohammed Moudjou, Davy Martin, Alexandra Busley, Tina Knäpple, Laetitia Herzog, Fabienne Reine, Nad’a Lepejova, Charles-Adrien Richard, Vincent Béringue, Human Rezaei

**Affiliations:** INRA, Université Paris-Saclay, UR892, Virologie Immunologie Moléculaires, Jouy-en-Josas, France; Dartmouth Medical School, USA, UNITED STATES

## Abstract

Mammalian prions, the pathogens that cause transmissible spongiform encephalopathies, propagate by self-perpetuating the structural information stored in the abnormally folded, aggregated conformer (PrP^Sc^) of the host-encoded prion protein (PrP^C^). To date, no structural model related to prion assembly organization satisfactorily describes how strain-specified structural information is encoded and by which mechanism this information is transferred to PrP^C^. To achieve progress on this issue, we correlated the PrP^Sc^ quaternary structural transition from three distinct prion strains during unfolding and refolding with their templating activity. We reveal the existence of a mesoscopic organization in PrP^Sc^ through the packing of a highly stable oligomeric elementary subunit (suPrP), in which the strain structural determinant (SSD) is encoded. Once kinetically trapped, this elementary subunit reversibly loses all replicative information. We demonstrate that acquisition of the templating interface and infectivity requires structural rearrangement of suPrP, in concert with its condensation. The existence of such an elementary brick scales down the SSD support to a small oligomer and provide a basis of reflexion for prion templating process and propagation.

## Author summary

Prions are self-propagating assemblies with all necessary and sufficient replicative information stored in the 3D structure of the misfolded form of PrP called PrP^Sc^. Since the emergence of the prion theory in the 80s, many attempts have been done to identify prion replicative information at molecular scale. Different models have been constructed based on a broad panel of experimental observations and some of them predict the existence of periodic elements constituting prion assemblies. Here, by using partial unfolding approaches, we trapped an oligomeric conformer that we called suPrP, which could constitute the elementary brick of prion assemblies. Once isolated, this elementary brick is devoid of infectivity. However, it becomes infectious once condensated into larger assemblies. The identification of the elementary PrP building block provides a new structural basis for understanding prion replicative information storage and spreading.

## Introduction

Transmissible spongiform encephalopathies (TSEs), or prion diseases, constitute a group of rare, fatal neurodegenerative diseases affecting humans and animals. Creutzfeldt-Jakob disease (CJD), Gerstmann-Sträussler-Scheinker syndrome (GSS) and fatal familial insomnia (FFI) are the most common forms of human TSEs. The prion theory, initially proposed to describe TSE pathogenesis [1], has recently been extended to a larger panel of neurodegenerative disorders resulting from protein misfolding and aggregation [2, 3]. While the aetiology of TSEs is associated with a template-assisted conformational change in the normal prion protein (PrP^C^) into an abnormal conformer (PrP^Sc^), the molecular mechanism of the templating process and its dynamics remain obscure. The most accepted theoretical mechanisms describing prion conversion remain Griffith’s autocatalysis hypothesis, proposed in 1967, and the seeding-elongation theory of Caughey-Lansbury (CL), proposed in 1995 [4, 5]. Although other models have been proposed, all these models include either Griffith or CL kernels [6–8] and do not describe the templating process in terms of molecular and structural events.

The field of protein folding has benefitted from more than five decades of conceptual and methodological development, whereas the exploration of amyloid assemblies and their mode of packing has only recently emerged but is of growing interest. For non-mammalian prions requiring poly N/Q repetition for segment assembly, the templating process occurs via induced-fit incorporation of the monomer at one extremity of the growing amyloid fibril [6]. This mechanism fits well with the structural model reported by Sawaya and collaborators [9]. Concerning mammalian prions, the formation of PrP^Sc^ assemblies is far from being elucidated. Many attempts to explore the architecture of prion assemblies in detail have been reported. While low-resolution structural approaches such as analyses of small angle X-ray scattering, hydrogen-exchanges and molecular dynamics based on experimental constraints have generated several models of PrP packing [10–18], these strategies have failed to describe the dynamics of the templating process. Indirect approaches, such as polymerization kinetic modelling and partial unfolding using ionic detergent, chaotropic treatment and temperature, have also failed to describe the templating process [19–25]. Therefore, the best mechanism that might be intuitively considered is the fitted-induced adjustment of PrP^C^ at both extremities of the PrP^Sc^ assembly [10] and the propagation of an allosteric state from PrP^Sc^ to PrP^C^ through an interface of interaction between these two conformers [26]. Although the structural models of PrP^Sc^ are divergent, they provide potentially useful information with regard to the architecture of the assemblies, such as the existence of a periodical repetition of multimeric PrP as an elementary base. For example, electronic diffraction performed on a 2D quasi-crystal revealed that the architecture of prion assemblies was based on the periodical repetition of a PrP trimer [15]. Another model based on a simulation of molecular dynamics performed on a H2H3 segment of PrP suggests the stacking of tetrameric PrP as the basis of amyloid fibre formation by the helix H2H3 segment [13]. Recently, Wadsworth and colleagues used electron tomography to show the existence of periodic elements constituting the extractive PrP^Sc^ tropo-filament [16]. The existence of such periodical repetition suggests the existence of a mesoscale organisation of PrP protomer in prion assemblies and immediately raises the question of the sequence of events during template-assisted conversion and of the potential structural polymorphisms that should exist among PrP^Sc^ conformational variants or strains.

To investigate the intimate architecture of infectious prion assemblies, we used sedimentation velocity and size exclusion chromatography methods to examine the quaternary structural transition during the partial unfolding of PrP^Sc^ assemblies at equilibrium using a chaotropic agent. During this partial unfolding process, we revealed that PrP^Sc^ assemblies are composed of oligomeric elementary bricks, referred to as suPrP, which are innocuous once isolated. The condensation of these molecules into larger assemblies results in the reacquisition of infectious properties through a conformational change at the protomer level, in concert with their condensation. Moreover, we demonstrated that PrP^Sc^ assemblies are in steady-state equilibrium with suPrP and could play a role in the spreading of the prion replication centre.

## Results

### Sequential unfolding and refolding of PrP^Sc^ reveals the existence of a small oligomeric subunit

**Fig 1:**
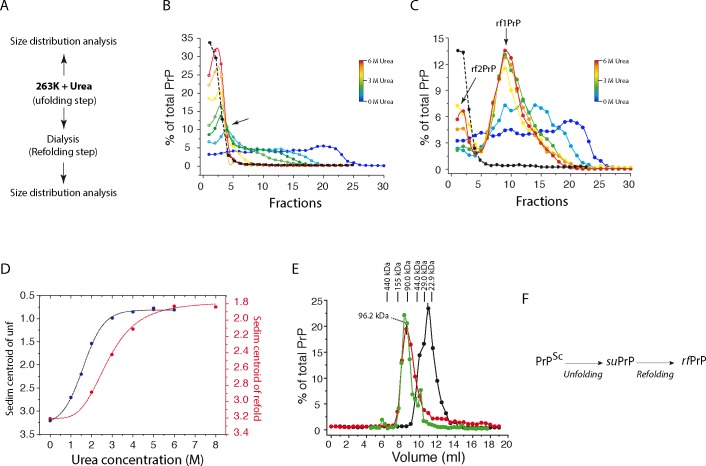
Sequential unfolding and refolding of PrP^Sc^ trapped an elementary conformer. (A) Overview of the procedure for unfolding and refolding native PrP^Sc^. (B) The effect of an increasing urea concentration on the quaternary structure of PrP^Sc^ was analysed based on SV. The superposition of the SV profile of PrP after urea treatment (the blue-to-red scale corresponds to 0 to 6 M urea) emphasizes the quaternary structure transition with an isobestic point (arrow) from highly polydisperse assemblies to a smaller and monodispersed conformer. The black line indicates the SV profile of PrP^C^ in 6 M urea (PrP^U^). (C) Similar experiments as in (B), with a refolding step via dialysis to remove urea (the blue-to-red scale corresponds to 0 to 6 M urea treatment before dialysis). The SV profile of PrP reveals the formation of two sets of assemblies termed rf1PrP and rf2PrP. The SV profile of PrP^C^ treated with urea and refolded via dialysis is represented as a black line with an intensity adjustment. (D) The evolution of the sedimentogram centroid of PrP^Sc^ unfolding (B) and refolding (C) as function of the urea concentration revealed a sigmoidal shape for the unfolding and refolding step, suggesting a cooperative phenomenon. (E) Size exclusion chromatography profile of partially purified suPrP^263K^ (in red), purified [31] suPrP^263K^ (in green) and purified PrP^C^ unfolded in 6 M urea (in black). As shown, partially purified and purified suPrP^263K^ presented an oligomeric structuration (for details, see Materials and Methods and appendix S2 and S3 Appendices). (F) scheme summarising the PrP^Sc^ unfolding process induced by urea and the formation of suPrP. This last refolds into rfPrP after urea removal by dialysis.

Unfolding of prions was performed by incubation with increasing concentrations of urea (from 0 to 6 M) for 60 min. After a solubilisation step involving dodecyl maltoside and sarkosyl, PrP size distribution was analysed by sedimentation velocity (SV) in an iodixanol gradient, as previously described [27, 28]. The SV buffer and brain homogenate contained identical concentrations of urea to prevent PrP refolding during the centrifugation step (Fig 1B). When the 263K-brain homogenate was treated with increasing concentrations of urea, a transition in the PrP quaternary structure was observed. This transition corresponded to a disassembly process, leading to the formation of small PrP conformers with a sedimentation peak centred on fraction 2 (Fig 1B). These conformers were designated as suPrP. At the resolution of the SV, suPrP co-sedimented with the unfolded state of PrP^C^ (PrP^U^) obtained after the treatment of uninfected hamster brain homogenate with 6 M urea (black curve in Fig 1B). The analysis of PrP sedimentogram evolution as a function of the urea concentration revealed the existence of an isobestic point, suggesting a two-state transition process within the range of 1 to 6M urea, without the accumulation of disassembling intermediates (for details see S1 Appendix). Moreover, the sedimentogram centroid (see Materials and Methods) as a function of the urea concentration presented a sigmoidal shape, indicating a cooperative disassembly process (Fig 1D, in black). After urea removal by dialysis prior SV, PrP spontaneously refolded into two sharp populations peaking in fractions 2 and 9, and designated rf2PrP and rf1PrP, respectively (Fig 1C). Furthermore, the formation of rfPrP assemblies was concerted with the disappearance of the small conformers generated during the unfolding step, as shown in Fig 1B. As for urea-induced disassembly, the evolution of the sedimentogram centroid as a function of urea presented a sharp sigmoidal shape, suggesting a highly cooperative refolding process (Fig 1D, in red). Comparatively, the refolding of PrP^U^ (i.e. PrP^C^ treated with 6M urea) by dialysis did not generate larger-sized PrP aggregates, indicating that rfPrPs are specific to PrP^Sc^ disassembling and reassembling.

The velocity sedimentograms suggest that suPrP is either monomeric as PrP^U^, or formed from either small-sized oligomers or low-density PrP assemblies. To estimate more precisely suPrP size, urea-treated and detergent-solubilized (as for SV) 263K brain homogenates were analysed by size exclusion chromatography (SEC). The SEC column was equilibrated with a running buffer containing 6 M urea (but no detergents) to avoid refolding during the separation. Of note, the presence of 6M urea in the running buffer disintegrate [29, 30] all lipoid micellar structuration potentially interfering with the intrinsic hydrodynamic size of suPrP. After SEC fractionation, the amount of PrP was estimated by western blot. While the urea-unfolded, purified PrP^C^ from uninfected hamster brains (see S2 Appendix) eluted at 11.3 ml, the elution volume of urea-treated suPrP from 263K-infected brain was approximately 8.2 ml, suggesting an oligomeric state (Fig 1E). According to our column calibration, suPrP would be in the range of a PrP trimer (with a standard error of ±1 protomer). Urea-treatment of PrP^Sc^ purified in the absence of detergents and associated lipids (S3 Appendix and [31]) led to a quasi-identical chromatogram (green curve in Fig 1E). Therefore, we can exclude a size overestimation of suPrP due to the contribution of a noncovalent interaction with lipids or detergent trace.

Collectively, these data indicate that PrP^Sc^ could disassemble into smaller oligomeric conformers, designated suPrP, showing a sedimentation peak centred on fraction 2. The formation of suPrP followed a two-state disassembly and cooperative process: *PrP*^*Sc*^ ⟶ *suPrP*. The refolding step leads suPrP to condense and generate rf1PrP and rf2PrP assemblies (Fig 1F).

### Presence of suPrP is a generic prion characteristic

**Fig 2:**
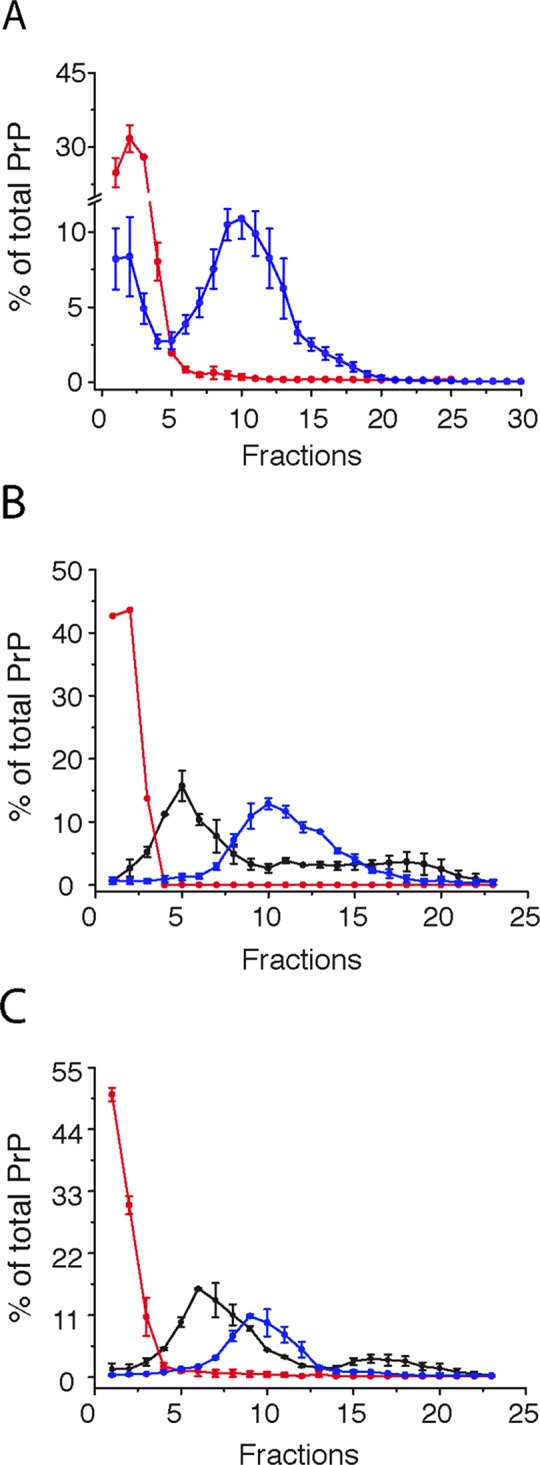
Formation of suPrP by different prion strains. SV profile of suPrP obtained after treatment of the homogenate with 6 M urea without dialysis (red line) and after refolding (blue line) for the 263K (A), T2^Ov-19K^ (B) and T1^Ov-21K^ (C) prion strains. For comparison, the SV sedimentograms for untreated infected brain samples are shown in black.

We next sought to determine whether the isolation of such suPrP conformers is a generic prion characteristic or restricted to the 263K prion strain. Therefore, two different prion strains, T1^Ov-21K^ and T2^Ov-19K^, obtained after adaptation of human CJD prions (cortical MM2 subtype) to tg338 mice [32, 33], were similarly treated with 6 M urea, followed by dialysis (Fig 1A and Fig 2A). As shown in Fig 2B and 2C, both T1^Ov-21K^ and T2^Ov-19K^ PrP^Sc^ unfolded into small objects that sedimented in the upper fractions of the gradient, as observed for suPrP^263K^, and refolded into larger-sized assemblies upon the removal of urea by dialysis. These observations joined those obtained using 263K PrP^Sc^ (Fig 1B and Fig 2A) and suggest the existence of a generic process leading to the formation of suPrP conformer endowed with the necessary and sufficient structural information to reassemble into rfPrP during the dialysis/refolding step. Due to biohazard restrictions, SEC of the suPrP^21K^ and suPrP^19K^ samples was not possible. However, the quasi-similar SV patterns of the suPrP^21K^ and suPrP^19K^ strains compared with suPrP^263K^ enabled the retention of a similar oligomer size as a first approximation.

### suPrP polymerization generates PK-resistant assemblies with templating and replicative activity

**Fig 3:**
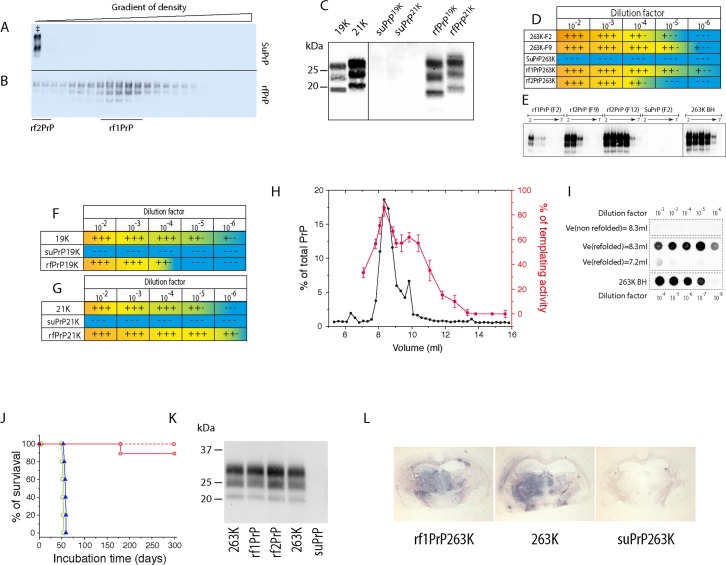
PK resistance, templating activity and infectivity of PrP^Sc^ during sequential unfolding and refolding. The PK sensitivity of suPrP (A) and rfPrP (B) was assessed after treating the SV fractions of the 6 M urea-treated 263K-brain homogenate without (A) or with a refolding step (B), respectively. ‡ indicates the PK resistance of the non-treated 263K-brain homogenate as a control. (C) For T1^Ov-21K^ and T2^Ov-19K^, SV fractions corresponding to their suPrP (pool of fractions F2 and F3 from Fig 2B and 2C) and fractions corresponding to rfPrP (fraction F10 from Fig 1B and 1C) were treated with PK. Lane 19K and 21K correspond to non-treated samples digested by PK. (D) Relative templating activity of SV-fractionated suPrP^263K^ (pool of fraction F1 to F3), rf1PrP^263K^ and rf2PrP^263K^ (respectively fractions F9 and F2 in Fig 1C). Fractionated assemblies were serially diluted before being used as a template in the PMCA reaction. The resulting products were analysed for PrP^res^ contents and typical western blot analysis of PMCA product in (E) (n≥9 independent assays for 263K prions). Similar experiments have been done for T2^Ov-19K^ and T1^Ov-21K^ prion strains (F and G and for typical western analysis of PMCA product see S7 Appendix). Positive (+++) and negative (—) detection are indicated using a yellow-to-blue gradient scale. The templating activities of fractions F2 and F9 from untreated 263K assemblies and fractions F5 and F6 from untreated T2^Ov-19K^ and T1^Ov-21K^, respectively, are shown for comparison. (H) Titration of the templating activity of suPrP^263K^ SEC fractions (see also Fig 1E, green line) after dialysis by PMCA (n = 3 independent assays). The templating activity is expressed as a percentage of that found in a 263K brain homogenate used as standard, as calculated by the ratio of the lowest concentrations resulting in a positive PMCA reaction (red curves). (I) Typical PMCA titration analysed by dot-blotting. Tenfold diluted dialysed SEC fractions corresponding to chromatogram peak (elution volume = 8.3) and for the base of the peak (elution volume = 7.2) were submitted to one round of PMCA and analysed for PrPres content by dot-blotting. The titration was compared to that obtained with 263K infected hamster brain homogenate (263K BH) as reference. (J) Survival of hamster PrP transgenic tg7 mice injected intracerebrally with SV-fractionated suPrP^263K^ (pool of fraction F1 to F3 in red line and pool of fraction F9 to F11 in dash red line), rf1PrP^263K^ (blue line) and rf2PrP^263K^ (green line) assemblies. PrPres^263K^ glycopattern (K) and neuroanatomical deposition (L) in the brains of hamster PrP mice inoculated with rf1PrP^263K^, rf2PrP^263K^ assemblies compared with 263K prions. The mouse used for suPrP^263K^ analysis was healthy and was euthanized at 200 days post inoculation.

To further assess the relationship between suPrP, rfPrP and the parental PrP^Sc^ assemblies, we compared their proteinase K resistance and templating activities *in vitro* or *in vivo*. All the fractions from the 263K-brain homogenate treated with 6 M urea before SV were PK-sensitive (Fig 3A), as compared to their non-urea treated counterparts. Similarly, the top fractions from the T1^Ov-21K^ and T2^Ov-19K^ brain homogenates treated under the same conditions were PK sensitive (Fig 3C), indicating that suPrP is PK sensitive. In contrast, the rfPrP species were truly PK resistant (Fig 3B and 3C), indicating a structural rearrangement of PrP protomers during the refolding and polymerization of suPrP into rfPrP. In the particular case of T1^Ov-21K^ and T2^Ov-19K^, rfPrP presented the parental 21K and 19K proteolytic signatures [32], respectively, in reference to unglycosylated PrP^res^ (Fig 3C). This observation suggests that during suPrP refolding into rfPrP, the tertiary structure of the PrP protomer refolds to acquire a proteolytic pattern similar to the native T1^Ov-21K^ and T2^Ov-19K^ strains. Thus, the structural determinant responsible for this differential proteolysis is maintained in the structure of suPrP.

To compare the specific templating activity of both suPrP and rfPrP with that of untreated PrP^Sc^ assemblies, we used protein misfolding cyclic amplification (PMCA). Serial ten-fold dilutions of suPrP enriched fractions (see also S4 Appendix), rfPrP-enriched SV fractions and fractions from the SV fractionation of untreated infected brain material (see Fig 2A–2C) were mixed with uninfected brain homogenate from hamster PrP transgenic (tg7 line) or ovine PrP mice (tg338 line) [28] and run for one round of PMCA for 48 hours [27]. The PMCA products were subsequently treated with proteinase K and analysed for PrP^res^ content by immunoblotting. All samples were normalized according to the total amount of PrP prior to PMCA to compare the specific templating activity per PrP unit. As shown in Fig 3D–3F, no PrP^res^ signal was detected after PMCA amplification of suPrP-enriched fractions from the three strains. In sharp contrast, a positive signal was observed after PMCA amplification of the rfPrP fractions up to a 10^5^−10^6^-fold dilution.

To exclude the contribution of cofactors or putative, remnant PrP^Sc^ assemblies to the refolding of inactive suPrP into active rfPrP after urea removal, size exclusion chromatography fractions collected after suPrP size-separation (Fig 1E, green curve) were dialyzed to remove urea and titrated for their templating activity by PMCA. As shown in Fig 3H, the templating activity (relative to that observed for 263K brain homogenate) perfectly correlates with suPrP size exclusion profile. According to PMCA titration, the templating activity of the suPrP peak was almost similar to that found in whole brain homogenate. Altogether, these observations suggest a quasi-total restoration of the templating activity after refolding of suPrP^263K^ into rfPrP. Moreover, the correlation between suPrP SEC profile with templating activity of these fractions after urea removal makes highly improbable the contribution of cofactors or remnant PrP^Sc^ seed to *suPrP* ⟶ *rfPrP* process.

We next compared the infectivity of suPrP^263K^ and rfPrP^263K^ conformers using an incubation time bioassay in hamster PrP transgenic mice [28]. A second objective of this experiment was to determine whether the 263K strain structural determinant (SSD) was fully conserved after suPrP^263K^ refolding and condensation into rfPrP^263K^. Pools of reporter tg7 mice were inoculated intracerebrally with 1:10-diluted, freshly prepared aliquots from the aforementioned fractions. As rfPrP formation occurs according to a cooperative process (as shown in Fig 1), the dilution of suPrP should significantly reduce the rate of rfPrP formation and enable trapping of the suPrP oligomer, even after the dilution of urea prior to inoculation. The tg7 individual survival time values are reported in Fig 3I. Only one out of five mice inoculated with suPrP-enriched fractions (fractions 1 to 3) developed a clinical prion disease and accumulated detectable PrP^res^ in the brain. The remaining mice, as the mice inoculated with intermediate fractions from the same gradient (fractions 9 to 11) remained free of symptoms and of PrP^res^ in the brain up to 350 days post-inoculation. Previous dose/survival time curves for 263K prions showed that at the limiting dilution (10^−6^), the mean survival time was established as approximately 100 days [28]. These observations indicate that the suPrP fractions exhibited extremely low levels of infectivity [34] that can occasionally trigger disease in reporter mice. In marked contrast, mice inoculated with the upper (fraction 1 to 3) and intermediate fractions (fraction 9 to 11) from SV-fractionated, urea-treated and dialysed 263K-brain material (i.e., enriched in rfPrP fractions) succumbed to disease after 56±1 and 58±1 days, respectively. In terminally sick mice, the brain PrP^res^ electrophoretic profile and neuroanatomical distribution of PrP^res^ were consistent and were similar to those observed in association with 263K prions (Fig 2J and 2K). A healthy mouse euthanized at 200 days post-inoculation with a suPrP fraction was negative for PrP^res^ accumulation (Fig 2J and 2K).

Collectively, these observations suggest that the suPrP conformer has generic properties, at least for the three prion strains examined here. The suPrP conformer is PK sensitive and exhibits low, if any, templating activity. However, once polymerized into rfPrP during the refolding step, the complex reacquires its templating capacity and the PrP^Sc^ strain structural determinant, as shown via bioassay experiments for rfPrP^263K^ and the electrophoretic typing for rfPrP^T1-Ov-21K^ and rfPrP^T2-Ov-19K^.

### Existence of equilibrium between PrP^Sc^ and suPrP

**Fig 4:**
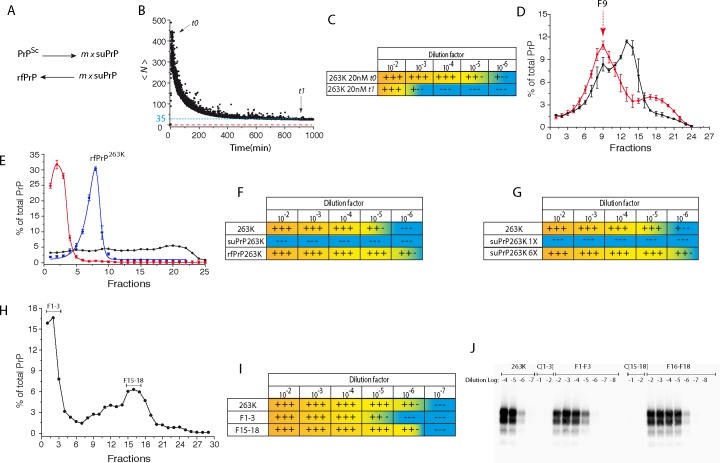
Equilibrium between PrP^Sc^ assemblies and suPrP. (A) The occurrence of a quaternary structural transition in PrP^Sc^ assemblies suggests the existence of an equilibrium between PrP^Sc^ and suPrP. This equilibrium could be displaced by dilution. *m* represents the number of suPrP molecules that condense to form rfPrP. (B) The depolymerization of purified 263K PrP^Sc^ was explored using a quick dilution method from 1 μM to 10 nM. The variation in the size of PrP^Sc^ assemblies was monitored by static light scattering. As PrP assemblies are highly heterodisperses, weight-average molecular weights were estimated (see S5 Appendix for more details). PrP size is expressed in terms of the weight-averaged number of PrP protomers (*<N>*) and reported as a function of time. The fact that the relaxation of *<N>* does not reach *<N> = 3* (estimated size of suPrP^263K^, dotted red line) suggests that the dilution factor (1 μM to 10 nM) is not sufficient to achieve total equilibrium displacement. (C) PMCA-templating activities of PrP^Sc^ assemblies at t0 and after size relaxation at the plateau (t1), as indicated with arrows in panel B and SI5 (n = 3). (D) Isopycnic concentration [36] of suPrP^263K^ (S4 Appendix). The 263K-infected brain homogenate was treated with 6 M urea, then subjected to isopycnic sedimentation and analysed for PrP contents (red line). The density distribution of untreated 263K PrP^Sc^ is shown for comparison (black line). Fraction 1 corresponds to lowest density (top of the isopycnic gradient), and fraction 27 corresponds to the highest density (bottom of the gradient). (E) The F9 fraction of the isopycnic gradient, indicated with an arrow in (D), was subjected to SV (in blue) (n = 5 independent assays). For comparison, the sedimentation profiles of untreated 263K assemblies and suPrP before isopycnic concentration are represented with black and red lines, respectively. (F) The F9 fraction subjected to SV and corresponding to rfPrP^263K^ was tested for templating activity in PMCA assays (n = 9). The templating activity of untreated 263K PrP^Sc^ and of suPrP (263K brain homogenate in 6M urea) was assessed in parallel runs. Refolding of suPrP into rfPrP was achieved using the urea dilution method. The 6-fold-concentrated suPrP fraction (suPrP^263K^ 6X) in 6 M urea was directly diluted 10-fold, either in PMCA reaction medium to estimate its templating activity (G) or in SV loading buffer prior to SV for size distribution analysis (H). The resulting fractions 1–3 and 15–18 were analysed for templating activity via PMCA (I). As controls in the PMCA reaction, either the non-concentrated suPrP^263K^ fraction in 6 M urea (suPrP^263K^ 1X, G) or 263K PrP^Sc^ (I) was used. The panel (J) shows representative PMCA product analysed by Western Blot.

The occurrence of a quaternary structural transition from PrP^Sc^ to suPrP and rfPrP at a low urea concentration (as low as 1 M urea, Fig 1B and 1C) strongly suggests the existence of equilibrium between rfPrP / PrP^Sc^ and suPrP under physiological conditions (i.e., without any need for urea treatment). This equilibrium state, schematized in Fig 4A, suggests that the PrP^Sc^ depolymerization rate, as first-order kinetics, is independent of the PrP^Sc^ concentration, while the condensation rate is suPrP concentration dependent. To document the existence of such equilibrium, in the absence of urea treatment, a high-speed dilution method was used to displace the equilibrium towards the formation of suPrP (S5 and S7 Appendixs). Purified 263K PrP^Sc^ assemblies (S3 Appendix) were rapidly diluted, and the relaxation of the mean average molecular size of PrP assemblies (*<N>*) was monitored as a function of time by static light scattering (Fig 4B). A decrease in the mean average size of PrP assemblies was observed, indicating equilibrium displacement of PrP^Sc^ assemblies towards smaller-sized objects. Three mutually exclusive hypotheses can explain the formation of small objects immediately after dilution. *i)* PrP^Sc^ may depolymerize into monomers. However, it is highly unlikely that in the absence of a chaotropic context, PrP^Sc^ assemblies generate monomers similar to PrP^C^ by simple dilution, while a 6 M urea treatment of PrP^Sc^ leads to the formation of the suPrP conformer. *ii)* PrP^Sc^ may spontaneously fragment through simple dilution and soft agitation. This event is also highly improbable. Indeed, comparison between the amplification titre via PMCA at t0 and at the plateau (t1) revealed a 3-log loss in PrP^Sc^ templating activity induced by dilution (Fig 4C). Fragmentation may generate more *de novo* templating seeds and should therefore lead to increased PMCA activity [35]. *iii)* The third hypothesis links PrP^Sc^ depolymerization to suPrP by equilibrium displacement during dilution. This hypothesis would be fully consistent with the reduction of PMCA templating activity. Total equilibrium displacement at a high dilution (1 μM to 20 nM) should provide an *<N>* value corresponding to the size of suPrP^263K^ (*<N>* = 3, dots red line in Fig 4B). The higher *<N>* value (*<N>≈*35) observed at the relaxation plateau suggested that the dilution factor was not sufficiently significant to achieve quasi-total equilibrium displacement, and larger assemblies still remained. Accordingly, complete loss of PMCA activity, as observed with suPrP^263K^, did not occur (for higher dilution factor see S1 Appendix, Fig S7).

To characterize the reverse condensation of suPrP^263K^ into rfPrP^263K^ assemblies (Fig 4A), we used two complementary approaches based on *suPrP* ⇌ *rfPrP* equilibrium displacement in favour of rfPrP. The first approach involved a isopycnic concentration method favouring an increase in PrP local concentration [36] in concert with urea removal (S6 Appendix). After isopycnic sedimentation of a 6M urea-treated 263K-brain homogenate, the majority of PrP was detected in fractions 6–16, with a peak being observed in fraction 9, corresponding to a low-density object (Fig 4D). Fraction 9 was collected and immediately subjected to SV. The resulting sedimentograms showed that isopycnically concentrated PrP was composed of one major population of larger-sized assemblies peaking in fraction 8 (Fig 4E). These assemblies exhibited similar specific PMCA templating activity per PrP unit compared to the 263K assemblies, suggesting that the isopycnic concentration of suPrP enabled the regeneration of rfPrP assemblies (Fig 4F).

The second approach was based on equilibrium displacement towards the formation of rfPrP after a decrease in the urea concentration by fast dilution. First, semi-purified suPrP^263K^ (S4 Appendix and Materials and Methods) in 6 M urea was concentrated six-fold by ultrafiltration (suPrP^263K^6X). Subsequently, suPrP^263K^ 6X was 10-fold diluted in PMCA reactional medium to dilute the urea concentration below 0.6 M. As shown in Fig 4G, dilution of urea restored the templating activity up to a 10^6^-fold serial dilution, when the non-concentrated suPrP^263K^ remained inactive. To analyse the effect of urea dilution on the PrP sedimentation profile, 6X suPrP^263K^ in 6 M urea was diluted 10-fold (for a final urea concentration of 0.6 M), and the quaternary structure was analysed by SV. As shown in Fig 4H, the sedimentogram revealed the formation of large PrP assemblies that were initially absent in non-concentrated suPrP^263K^. Furthermore, analysing the templating activity of fractions 1 to 3 and 15 to 18 by PMCA (Fig 4I) revealed an activity similar to 263K prions. Thus, this second approach based on dilution of the urea concentration below 1 M revealed the spontaneous condensation of semi-purified suPrP^263K^ into rfPrP.

Collectively, the equilibrium displacement experiments demonstrated that rfPrP / PrP^Sc^ assemblies are in dynamic equilibrium with suPrP. Equilibrium displacement by dilution decreases both the size of PrP^Sc^ assemblies and the templating propensity after complete relaxation. However, the equilibrium could be shifted towards the formation of rfPrP / PrP^Sc^ assemblies with templating activity by urea dilution or suPrP concentration.

## Discussion

Since the emergence of the prion theory and the identification of PrP^Sc^ assemblies as the main support for pathological information, the dynamics and level of organization of these assemblies have remained elusive. By using progressive unfolding and refolding approaches coupled to techniques probing quaternary structure transition, we have examined the mode of organization of PrP^Sc^ assemblies from three different prion strains, at the mesoscopic level. We showed that the quaternary structure of prion assemblies result from the condensation and rearrangement of non-infectious small oligomeric subunit (i.e., suPrP) harbouring the strain structural determinant. The existence of such subunit as an elementary brick and its dynamics during the formation of prion assemblies have important consequences at the level of templating mechanisms, structural determinisms of the prion strain and the spreading of prion replication centres.

### PrP^Sc^ assemblies disassemble into suPrP elementary bricks

Urea-induced PrP^Sc^ disassembling process generates a PK-sensitive and non-infectious conformer (suPrP), which upon refolding, exhibits the main structural determinants of extractive PrP^Sc^. The isobestic point deduced from the superposition of the PrP SV sedimentograms at increasing urea concentrations (within range of 1M to 6M urea, Fig 1B) and the sigmoidal shape of the sedimentogram centroid (Fig 1D) indicate that the formation of suPrP^263K^ occurs according to a cooperative two-state unfolding process (*PrP*^*Sc*^ ⟶ *suPrP*). This two-state mechanism implies that urea-induced depolymerization leads to the accumulation of a unique conformer (suPrP), without any significant accumulation of other reaction intermediates.

The use of isopycnic concentrations and refolding by urea dilution demonstrated the kinetic entanglement between suPrP^263K^ and rfPrP^263K^. As is the case for condensation/polymerization reactions, the rate of this process depends non-linearly on the concentration of the reactant (i.e., suPrP). This dependency of the rate of rfPrP formation on the suPrP concentration is also at the origin of the hysteresis and shift observed between the two-centroid sedimentogram sigmoids reported in Fig 1D. Indeed, the existence of hysteresis between the two curves implicates that the pathway of suPrP formation (*PrP*^*Sc*^ ⟶ *suPrP*) differs from the rfPrP one (*suPrP* ⟶ *rfPrP*). The comparison between urea1/2 of unfolding ($U_{1/2}^{PrPSc\to suPrP}$) and refolding ($U_{1/2}^{suPrP\to rfPrP}$) shows a difference of 1.1 M urea. This difference indicates that the cooperativity of *suPrP* ⟶ *rfPrP* process is higher than the *PrP*^*Sc*^ ⟶ *suPrP*.

**Fig 5:**
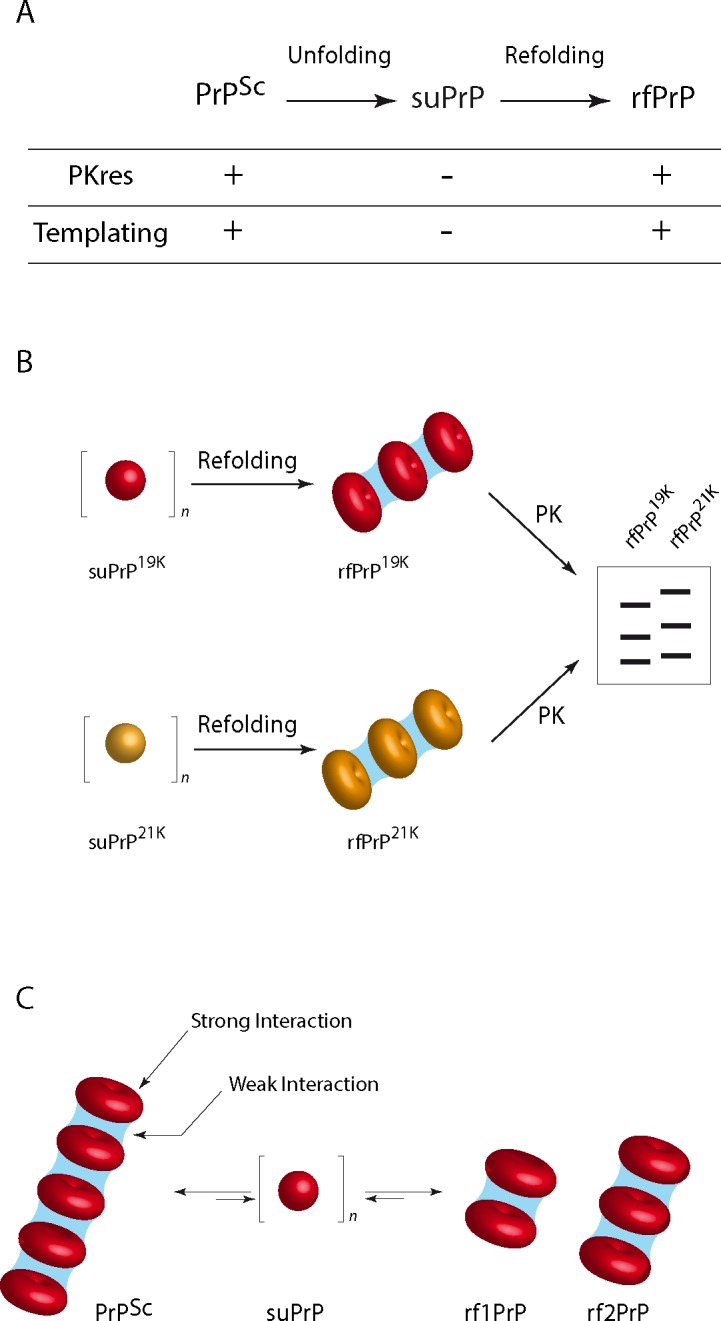
The role of suPrP in the dynamics of PrP^Sc^assemblies. (A) Evolution of PK resistance and templating propensities of different types of PrP assemblies obtained after sequential unfolding and refolding of the parental prion. PrP^Sc^ is the native prion; suPrP is the elementary oligomeric PrP subunit; and rfPrP is the refolded conformer formed after the polymerization of suPrP. The process of conversion of suPrP into rfPrP requires a conformational change in the PrP protomer of suPrP (represented here as a sphere) to form infectious and PK-resistant assemblies (represented as stack of torus). (B) The conserved differential proteolytic pattern of rfPrP^T1-Ov-21K^ and rfPrP^T2-Ov-19K^ suggests that their respective suPrPs (represented respectively as yellow and red spheres) exhibit distinct conformations. During the refolding step (C), two modes of organization contribute to the cohesion within PrP^Sc^ assemblies. Weak interactions (in blue) are involved in maintaining the overall quaternary structure by stacking suPrPs, when strong interactions are involved in the cohesion of PrP protomers in suPrP oligomers. The weakness of the interactions interlinking suPrP means that PrP^Sc^ assembly and disassembly are highly dynamic events, even in the absence of a chaotropic agent, and free suPrP could exist in equilibrium with infectious assemblies.

According to standard phenotypic criteria (PrP^res^ electrophoretic signature, incubation duration, PrP^res^ neuroanatomical distribution), the strain structural determinant (SSD) of 263K prion assemblies is fully conserved throughout the process of disassembling into suPrP and refolding into rfPrP. Similarly, the conserved, specific proteolytic signatures of rfPrP^T1-ov-21K^ and rfPrP^T2-ov-19K^ strains after the refolding of their respective suPrPs also indicates that the structural determinant responsible for differential proteolysis is contained within suPrP^T1-Ov-21K^ and suPrP^T2-Ov-19K^ (Fig 5B). Taken together, these observations suggest that suPrP is an elementary brick containing all the folding and structural information that is necessary and sufficient for reassembly into *bona fide* infectious rfPrPs with strain properties of the parental prion. Encoding of the SSD within the suPrP oligomeric structure suggests that this molecule can adopt different conformations. For practical reasons, we were unable to estimate the precise size of suPrP^T1-Ov-21K^ and suPrP^T2-Ov-19K^. Thus, it remains to be determined whether this structural polymorphism resides not only in the tertiary structure of the PrP protomer but also in the number of protomers forming the suPrP oligomers (i.e., quaternary structure of suPrP).

Notably, while rfPrP and extractive PrP^Sc^ exhibit a similar SSD, their respective size distribution patterns differ. The variability in the size distribution of rfPrP and its size homogeneity compared with extractive PrP^Sc^ assemblies have been well described based on the theory of condensation/polymerization. This last being highly stochastic and sensitive to local fluctuation [37]. Thus, the broader size distribution of extractive PrP^Sc^ assemblies should be considered with regard to biological environmental fluctuations in which PrP^C^ is converted into PrP^Sc^, whereas the *in vitro* refolding of suPrP into rfPrP occurs in a physically controlled environment.

### Existence of multiscale organization in different PrP^Sc^ structural models

Using SEC, we demonstrated that suPrP^263K^ is oligomeric and might correspond to a PrP trimer (we attributed +/-1 monomer due to SEC technic and calibration method). This last observation contrasts with previous experiments which suggest the existence of monomeric β-enriched conformer during guanidine-hydrochloride treatment [19]. Strikingly, suPrPs from 263K, T1^Ov-21K^ and T2^Ov-19K^ resist up to 8 M urea, whereas the overall PrP^Sc^ quaternary structure start to disassemble at ~1 M urea. These observations suggest that 263K, T1^Ov-21K^ and T2^Ov-19K^ assemblies have at least two levels of organization involving protomer interactions of different strength. One is highly sensitive to urea, suggesting ‘weak’ intermolecular interactions. Disruption of these interactions results in the formation of suPrP species. The second type of arrangement is highly stable, resistant to chaotropic unfolding, and maintains the oligomeric cohesion of suPrP (Fig 5C). The existence of these two modes of packing therefore involves at least two distinct PrP domains, one domain involved in the formation of the oligomeric elementary subunit and a second domain involved in its condensation. By using protease finger-print coupled to epitope-mapping, Kocisko and collaborators revealed that a 16kDa fragment, including the region of 143–154, remained the ultimate domain of PrP resisting to guanidine treatment [38]. This domain may constitute a region involved in the formation of suPrP.

Is such multiscale organization architecture (or at least a signature thereof) observed in the current PrP^Sc^ structural models that have recently been elaborated? The structural interpretation of our observations do not plead for PrP^Sc^ assemblies formed by the unique juxtaposition of parallel element in-registered β, as the interactions between each protomer are energetically equivalent [12, 14]. Two other models show different types of interactions to form the fibril core and to stack the subunits [15, 39, 40]. Daggett and co-workers proposed a β-spiral model based on the repetition of a trimeric core [39]. Even if this hypothesis is consistent with the data obtained in the present study, the high content of α-helices in this model is challenged by studies showing that PrP^Sc^ does not include an α-helix secondary structure [14]. The β-helix model presents an interesting organization based on a trimer of β-helices, despite the presence of α-helix contents [40]. A recent cryo-electron microscopy structural characterization [17] of GPI-anchorless PrP^Sc^ (PrP^Sc^ GPI^-^) led to refinement of the β-solenoid model, which now resembles the β-helix model without any remnant α-helix. This model strongly supports the existence of dimers resulting from head-to-head and tail-to-tail contacts. These dimers can be interpreted as the elementary bricks of the fibrils. Moreover, PrP^Sc^ GPI^-^ assemblies comprise two fibrils, implying lateral interactions between each fibril. Thus, it is reasonable to suggest that the elementary brick of these fibres is a dimer-dimer or a tetramer, depending on the strength of the interactions. In the present study, we did not obtain any information on whether suPrP is the elementary brick of a protofibril or fibre, even if an axis with 3-fold symmetry would favour a fibre brick over a protofibril brick [41]. The estimated size of suPrP, based on the limited precision of size exclusion chromatography, is at least within the range of what is observed in these structural models. Reflecting the resolution of the size determination experiments, we cannot exclude the idea that size estimation corresponds to a mixture of dimers and tetramers, as suggested by the recent structural model [17]. These data are consistent with these last two models. Moreover, the possibility that different prion strains differ in the size and structure of their elementary bricks is not excluded.

### Infectivity and PK resistance require structural rearrangements of suPrP during condensation

As demonstrated either by PMCA or bioassay, suPrP oligomers have very low templating activity and infectivity, if any. The immediate consequence of these observations is the absence of remnant seeds catalysing *suPrP* ⟶ *rfPrP* process. Indeed, these two techniques are highly sensitive, detecting seeds present in a 263K-infected brain homogenate diluted up to 10^7^- and 10^6^-fold, respectively [27, 28]. One important question that could arise is why suPrP does not completely refold into rfPrP after dilution at the time of mouse inoculation or PMCA titration? The reason of the absence of significant refolding could be the concentration dependency of the formation rate of rfPrP as the *suPrP* ⟶ *rfPrP* is a multimolecular process and therefore highly concentration dependent. This concentration dependency of *suPrP* ⟶ *rfPrP* refolding has been demonstrated as well by the isopycnic concentration method as by PMCA using 6-fold concentrated suPrP^263K^ (Fig 4G–4I).

We show that suPrP and infectious assemblies (rfPrP and PrP^Sc^) exhibit clearly different biochemical and biological properties, such as resistance to PK and templating activity/infectivity, suggesting a profound structural rearrangement of suPrP during polymerization into infectious assemblies (Fig 5A). Therefore, unique condensation of suPrP into rfPrP, without significant structural modification is not supported by the data obtained in the present study. Gain of PK resistance is attributed to a polypeptide backbone rearrangement, or formation of quaternary structure restricting protease accessibility. Indeed, PK sensitivity is not a question of the size of the assemblies if we consider a strict periodic repetition of an element. Moreover, the acquisition of infectivity and templating activity implies the creation of a templating interface in concert with the polymerization process of suPrP into rfPrP. These two observations strongly suggest that during polymerization, protomers constituting suPrP undergo at least a tertiary structural rearrangement leading to the formation of PK-resistant and infectious assemblies. It is not surprising that condensation/polymerization occurs in concert with a major structural modification. The best example is the formation of amyloid assemblies by disordered domains of amyloidogenic proteins or even PrP^C^ to PrP^Sc^ conversion. From a physical point of view, this deep structural rearrangement is related to non-linearity of energy as function of size [26, 41].

The lack of infectivity of suPrP oligomer, should be put in perspective of the observations made by Silveira and collaborators who determined that 263K PrP^res^ oligomers smaller than a pentamer were virtually devoid of infectivity/templating activity [42]. Based on considering suPrP as a trimer (+/- 1 monomer) and the requirement of a condensation process to generate infectious assemblies, it can be expected that the necessary and sufficient structural rearrangements could occur with a suPrP dimer (i.e., a hexamer +/- 2 protomers of PrP). The suPrP dimer would therefore constitute the minimal size of PrP assemblies with replicative properties and could correspond to the rf1PrP observed in Fig 1C. This last could be poorly separated from PrP^C^ and suPrP due to the limited resolution of SV.

### Depolymerization instead of fragmentation may spread the replication unit

The destabilization of 263K PrP^Sc^ assemblies by urea is a highly cooperative process (as shown in Fig 1B and 1C), which after refolding, leads to the formation of rfPrP. The genesis of rfPrP is not a consequence of sequential depolymerization of initial PrP^Sc^ assemblies, but instead results from the condensation of PK-sensitive and non-infectious suPrP conformers. As shown in Fig 4B based on the fast dilution method, 263K PrP^Sc^ assemblies spontaneously depolymerize towards suPrP. Indeed, according to these results, it is highly improbable that the depolymerization process generates either monomeric PrP^C^ or PrP^Sc^ fragments. The fact that the relaxation of <N> tends asymptotically towards a value above a trimer suggests the existence of large object assemblies and, thus, partial equilibrium displacement at 10 nM (for lower concentration see S7 Appendix). The condensation of suPrP into rfPrP concerted with the reacquisition of templating activity has also been demonstrated using an isopycnic concentration method and through the refolding of semi-purified suPrP^263K^ via urea dilution. The existence of this process confirms the existence of a dynamic equilibrium and challenges the widespread idea of the need for an active fragmentation process to spread the prion replication unit and induce exponential growth [43–45]. Indeed, if we consider the equilibrium between PrP^Sc^, suPrP and rfPrP, a simple depolymerization/condensation process could be sufficient to spread the replicative unit through the release of suPrP, followed by its Brownian diffusion and condensation into rfPrP (Fig 5C). Therefore, the elementary brick of PrP^Sc^ assemblies may also play a role in the process of spreading the vector and circumventing fragmentation that is widely referred to by analogy to yeast prions [46].

### Conclusions

The existence of at least two levels of organization within prion assemblies implies that the conversion process could result from the assembly of an oligomeric subunit, referred to here as suPrP. The existence of an oligomeric subunit raises a question concerning the sequence of events during the templating process and how this elementary brick could be *de novo* formed and be integrated into assemblies. According to our study, we may propose two polymerization mechanisms that are not mutually exclusive but require further investigations. Indeed, either the template induces the formation of the trimer, or the trimer already exists as a minority species that is readily taken up by the template. The second hypothesis implies that in a cellular context, PrP^C^ and suPrP are in equilibrium, with a disadvantage for suPrP species. The presence of prion assemblies could displace this equilibrium by integrating suPrPs.

The particularly high stability of suPrP against denaturation raises the question of why PrP^C^ does not spontaneously switch to this thermodynamically stable suPrP conformation and therefore into infectious assemblies? In addition to the evocation of cofactors or a contribution of housekeeping proteins to prevent this spontaneous conversion, the oligomeric state of suPrP means that its formation is highly dependent on the PrP^C^ concentration and therefore kinetically controlled by the cell. The high stability of suPrP has also a repercussion for all drug design strategies aimed at disrupting or inactivating prion assemblies. Indeed, such drugs should exhibit an extremely high affinity to disrupt or trap the prion elementary brick, which resists up to 8 M urea.

## Materials and methods

### Ethics statement

All animal experiments were approved by the INRA/AgroParisTech Ethics Committee (Comethea) (permit number (V.B) 15/56). Euthanasia of animals was performed by cervical column disruption. The animals’ brains were rapidly removed and stored at -80°C for further analysis. All the experiments involving animals were carried out in strict accordance with EU directive 2010/63

### Brain homogenates and PrP^Sc^ purification

A stock of 263K-infected hamster brain homogenates (20% wt/vol. in 5% glucose) was prepared using nine infected Syrian hamsters at the terminal stage of disease and was stored at -20°C. Stocks of brain homogenates from non-infected hamsters were prepared in a similar manner. The T1^Ov-21K^ and T2^Ov-19K^ strains were isolated after serial transmission of a human sCJD brain extract (MM2, rare cortical form) to ovine PrP tg338 mice [32]. Three brains were collected at the terminal stage of disease and homogenized at 20% wt/vol. in 5% glucose to obtain the brain extract stock.

Purified PrP^Sc^ from hamster brains infected with the 263K prion strain was prepared according to the protocol of Baron and collaborators [31] (for more details, see S3 Appendix). Briefly, this protocol leads to obtain purified infectious 263K prion assemblies without the presence of significant amount of noncovalent associated lipids that could affect light scattering and SEC analysis.

### Urea treatment and refolding by dialysis

The urea concentration in 20% prion-infected brain homogenates has been adjusted using either urea crystal dissolution or appropriated dilution of 8M urea stock solution. Subsequently, the urea treated brain homogenate was incubated at 37°C for 1 h. When specified, urea was removed at 25°C by dialysis against 30 mM Tris/HCl, pH 8.0 for a minimum of four hours, using a micro-dialysis (cut-off membrane 14 kDa). Prior further analysis, brain homogenates were solubilized by adding an equal volume of a solubilization buffer [28] containing 4% (w/v) dodecyl-β-D maltoside. After incubation for 30 min at 4°C, sarkosyl (N-lauryl sarcosine) was added to give a final concentration of 2% (w/v) in the samples. The incubation was pursued for a further 30 min at 4°C.

### Sedimentation velocity fractionation, PK treatment and size exclusion chromatography analysis

SV experiments were performed as previously described [28]. Briefly, 150 μl of solubilized samples (Cf. supra) was carefully loaded on a 4.8 ml continuous 10–30% iodixanol gradient (Optiprep, Sigma-Aldrich), with a final concentration of 25 mM HEPES pH 7.4, 150 mM NaCl, 2 mM EDTA, 0.5% Sarkosyl. For PrP^Sc^ unfolding (Fig 1B) gradients media have been adjusted at the requested urea concentration. The gradients were centrifuged at 285 000 g for 45 min in a swinging-bucket SW-55 rotor. The collected fractions were analysed by western blotting using a biotinylated anti-PrP Sha31 monoclonal antibody after migration using SDS PAGE Criterion TGX gels. Western blot signal intensities were quantified using GeneTools software and converted into arbitrary units after normalization. For all SDS-PAGE analyses, a fixed quantity of human recombinant PrP was employed to calibrate the PrP western blot signals in different gels. The PK resistance of fractions was tested by the addition of PK at a final concentration of 50 μg/ml for 1 hour at 37°C prior to western blot analysis, as described above. The sedimentogram centroid, which determines the barycentre of a curve, was calculated using $\frac{\sum_{1}^{n}f(n).n}{\sum_{1}^{n}f(n)}$ with *f*(*n*) as the percentage of total PrP and the fraction number, *n*.

SEC analysis was performed using an AKAT-100 purifier FPLC with an S200 Increase 10/300 GL column from GE. The running buffer was 50 mM Tris/HCl, pH 7.2, containing 6 M urea. After sample injection, the flow-through of the column was fractionated every 500 μl. SV fractions containing suPrP (fractions 1 to 3) were recovered in 50 mM Tris/HCl, pH 7.2, containing 6 M urea, prior to injection into the SEC column. The presence of 6M urea in the running buffer leads to disintegrate all lipoid micellar structure [29, 30]. The PrP levels per fraction were estimated by western blotting, as for SV. As internal references for molecular weight estimation, recombinant purified PrP and PrP^C^ purified from a healthy tg338 mouse brain (S4 Appendix) were recovered in 50 mM Tris/HCl, pH 7.2, containing 6 M urea (the same buffer employed for suPrP). The templating activity of SEC fractions was determined by PMCA after micro-dialysis overnight of fractions against 50 mM Tris/HCl, pH 7.2.

### Fast dilution, isopycnic concentration and urea dilution

A stock solution of 1 μM purified PrP^Sc^ 263K ([31], Cf. supra) was diluted at a 10 nM final concentration in 20mM MOPS, pH 7.2 via fast injection into a cyclic-flow circuit coupled with a multi-wavelength static light scattering device (S2 Appendix). The weight-average molecular weight (<Mw>) was estimated using Rayleigh’s equation expanded to the second viral term for a heterogeneous macromolecule solution.

For polymerization induced using the isopycnic concentration method, a 263K brain lysate was first treated with 6 M urea at 37°C for 1 h, then mixed with 880 μl of 50% iodixanol, 25 mM HEPES, 150 mM NaCl, 5 mM EDTA, and 0.5% sarkosyl to obtain a final concentration of 40% iodixanol and subsequently subjected to isopycnic sedimentation (for a detailed description, see S4 Appendix). This mixture corresponds to the third pillow of a 10–60% discontinuous gradient (with 25 mM HEPES, 150 mM NaCl, 5 mM EDTA, and sarkosyl at a 0.5% final concentration). Next, the gradient was ultracentrifuged at 116,000 g for 17 h at 4°C in a swinging-bucket SW-55ti rotor, prior to fractionation into 30 fractions of 165 μl. The PrP distribution pattern was determined as for SV. Fraction F9, corresponding to rfPrP, was reloaded for SV analysis, for size analysis or PMCA assays.

For polymerization induced by dilution with 6 M urea, semi-purified suPrP^263K^ in 6 M urea (for detailed see S5 Appendix) was concentrated 6-fold via ultrafiltration (Sartorius vivaspin 500) with a membrane cut-off of 30 kDa. The concentrated product was then diluted 10-fold in the miniaturized bead-PMCA buffer used to prepare serial dilutions.

### Miniaturized bead-PMCA assay

The specific templating activity of the isolated PrP assemblies was measured using the mb-PMCA technique [27]. Briefly, *ex tempore*, serial ten-fold dilutions of the fraction containing the conformer of interest were immediately mixed with the brain lysate (10% wt/vol.) from healthy tg7 mice, as a substrate for 263K-seeds, or from healthy tg338 mice, for the two T1^Ov-21K^ and T2^Ov-19K^ strains. One round of 96 cycles (30 s sonication at 220–240 Watts, followed by 29.5 min of incubation at 37°C) was performed in a 96-well microplate, using a Q700 sonicator (Delta Labo, Colombells, France). Aliquots of the amplified samples were PK digested at a final concentration of 115 μg/ml with 0.6% SDS for 1 h at 37°C prior to immunoblot analysis, as described above.

### Mouse bioassay

A pool of 3 fractions containing the PrP conformer of interest was extemporarily diluted ten-fold in 5% glucose and immediately inoculated by intracerebral route to reporter tg7 mice (20 μl per pool of fraction, n = 5 mice per pool). At the terminal stage of the disease or at 350 days post-inoculation, the mice were euthanized, and their brains were removed and analysed for PrP^res^ content using the Bio-Rad TsSeE detection kit [7] prior to immunoblotting, as described above. The survival time corresponds to the number of days from inoculation to euthanasia. A histoblotting procedure (neuroanatomical distribution of PrP^res^) was performed on brain cryosections (8–10 μm thick) transferred to Superfrost slides, using a 3F4 anti-PrP antibody, as previously described [28].

## Supporting information

S1 Appendix Relation between isobestic point and two-state PrP^sc^ disassembly. (DOCX)

S2 Appendix Purification of PrP^C^ from healthy tg338 mice brain. (DOCX)

S3 Appendix PMCA templating activity of purified 263K PrP^Sc^ assemblies. (DOCX)

S4 Appendix The suPrP semi-purification procedure and the effect of suPrP concentration into rfPrP refolding. (DOCX)

S5 Appendix Fast kinetic dilution and determination of size variation of PrP assemblies by light scattering intensity and templating activity assay by PMCA. (DOCX)

S6 Appendix Isopycnic concentration and refolding of suPrP into rfPrP assemblies. (DOCX)

S7 Appendix The *PrP*^*Sc*^ ⇄ *i*. *suPrP* equilibrium displacement kinetic study by PMCA and the effect of high dilution. (PDF)
