# Supplementary material for: Reversible unfolding of infectious prion assemblies reveals the existence of an oligomeric elementary brick
Source: PLoS Pathog. 2017 Sep 7;13(9):e1006557. doi: 10.1371/journal.ppat.1006557 (PMC5589264; doi:10.1371/journal.ppat.1006557)
Supplement: S1 Appendix — (DOCX) [file ppat.1006557.s001.docx]

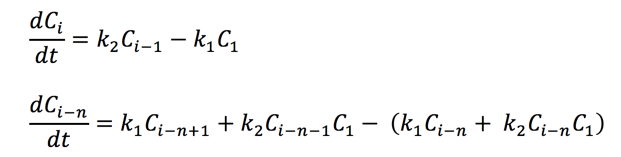

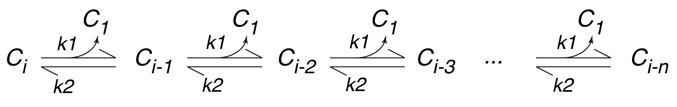

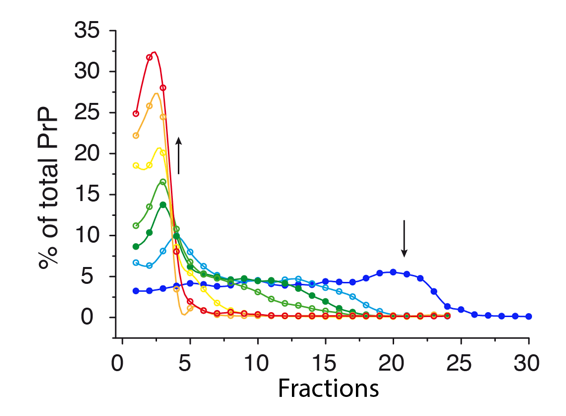


B

A

D

C


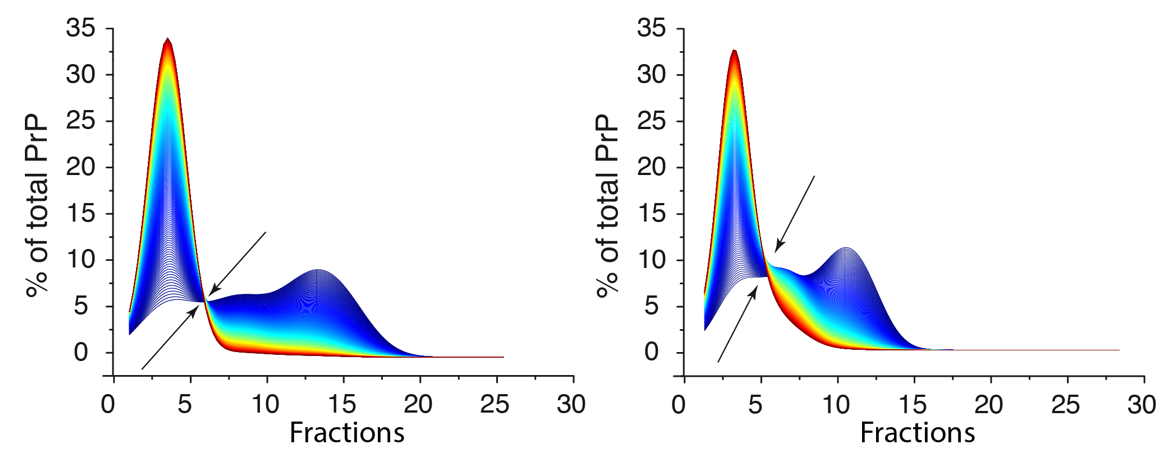


## S1 Appendix: relation between isobestic point and two-state PrP^sc^ disassembly.

The existence of an isobestic-point in the evolution of an observable along the extent of reaction, here sedimentograms describing the evolution of PrP^Sc^’s size distribution as function of urea (**A**), has important implication. Indeed, if we assume that in the disassembly process of PrP^Sc^ induced by the increasing chaotropic concentration a thermodynamic equilibrium has been reached for each urea concentration, the existence of an isobestic-point explicitly implicates a two-state process. The two-state process means that the reaction can be summarize by an elementary equilibrium such as $nA\rightleftharpoons A_{n}$. The immediate implication of this minimalist reaction is that the process involved only two-state of the reactant and along the extent of reaction any reactional intermediate exists. The extension of this assumption to PrP^Sc^ disassembly induced by urea as shown in figure 1B could be not so trivial as the non-treated PrP^Sc^’s size distribution clearly reveals the existence of broad distribution which corresponds to the superimposition of sedimentation of discreet object of different size. Therefore, it appears that these discreet objects forming PrP^Sc^’s assemblies could depolymerise either in a sequential way or by spontaneous disintegration with both of the process leading to the formation of conformers rising as a peak at fraction 2 with the existence of an isobestic point.

The analysis of sedimentograms as function of urea concentration reported on figure 1B, reveals immediately that we can exclude a global disintegration process (**A**). Indeed, the quaternary structure transition reveals that first large assemblies start to depolymerise. Thus it remains sequential depolymerisation as second possibility. To illustrate the two-state depolymerisation, a sequential depolymerisation process with 8 objects has been designed (**B**, with *i=8*). Differential equations relative to the process have been numerically solved for a given *k_1_* and *k_2_* till the equilibrium. To simulate the effect of urea, we performed simulation by increasing in a sigmoidal way *k_2_* as function of urea concentration ($k_{2}\propto[Urea]$). When any assemblies are accumulated during the depolymerisation a well define isobestic point can be observed (**C**). If one of the assemblies has a *k_1_* or *k_2_* slightly different the isobestic point is lost due to the accumulation of reactant (**D**).

Therefore, the existence of the isobestic point strongly suggest that the PrP^Sc^ depolymerisation follow a two-state model with one hand PrP^Sc^ as *C_i>3_* and *C_1_* as suPrP.
