# Supplementary material for: Reversible unfolding of infectious prion assemblies reveals the existence of an oligomeric elementary brick
Source: PLoS Pathog. 2017 Sep 7;13(9):e1006557. doi: 10.1371/journal.ppat.1006557 (PMC5589264; doi:10.1371/journal.ppat.1006557)
Supplement: S2 Appendix — (DOCX) [file ppat.1006557.s002.docx]

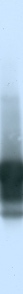


**38**

**28**

**17**

**50**

**75**

**100**


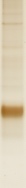


**S2 Appendix: Purification of PrP^C^ from healthy tg338 mice brain.**

PrP^C^ was purified from tg338 mice brain overexpressing the ovine VRQ allele by three successive chromatographic steps. 10 % brain lysate was prepared in Lysis Buffer (LB): 50 mM Tris-HCl pH 7.4, 1% Triton-X100. After clarification by centrifugation at 10.000g during 5 min, brain lysate was injected to a 5 mL HiTrap-SP-Sepharose cation exchange column using AKTA-100 Purifier chromatography system (GE-Heathcare). After extensive washing with LB, bound proteins were eluted with salt gradient up to 1M NaCl in LB at 1mL/min of flow rate during 20 min. Collected fractions were analysed by silver nitrate and western blotting with anti-PrP mAb Sha31. The interesting fractions were pooled and loaded onto a 5 mL HiTrap-immobilized-metal affinity column (IMAC) charged with 0.2 M nickel sulfate and equilibrated with LB containing 5 mM Imidazole and 150 mM NaCl. The unbound proteins were discarded and the column was washed with LB/5mM Imidazole. A 20 min gradient from 5 mM to 200 mM Imidazole in LB containing 150 mM NaCl was then applied to elute the PrP^C^. Fractions were analysed by silver staining and western blot for PrP^C^. The PrP^C^ containing fractions were pooled and injected to 5 mL HiTrap-Heparin affinity column equilibrated with LB. The column was extensively washed with LB and a salt gradient up to 1M NaCl in LB was applied to the column during 20 min at flow rate of 1mL/min. The collected fractions were analysed by silver nitrate staining and western blot for PrP^C^ detection. An example of highly enriched PrP^C^ fraction from the heparin column step is shown after silver staining (left panel) and western blotting (right panel). Molecular mass marker are indicated on the left.
