# Supplementary material for: Reversible unfolding of infectious prion assemblies reveals the existence of an oligomeric elementary brick
Source: PLoS Pathog. 2017 Sep 7;13(9):e1006557. doi: 10.1371/journal.ppat.1006557 (PMC5589264; doi:10.1371/journal.ppat.1006557)
Supplement: S3 Appendix — (DOCX) [file ppat.1006557.s003.docx]

**
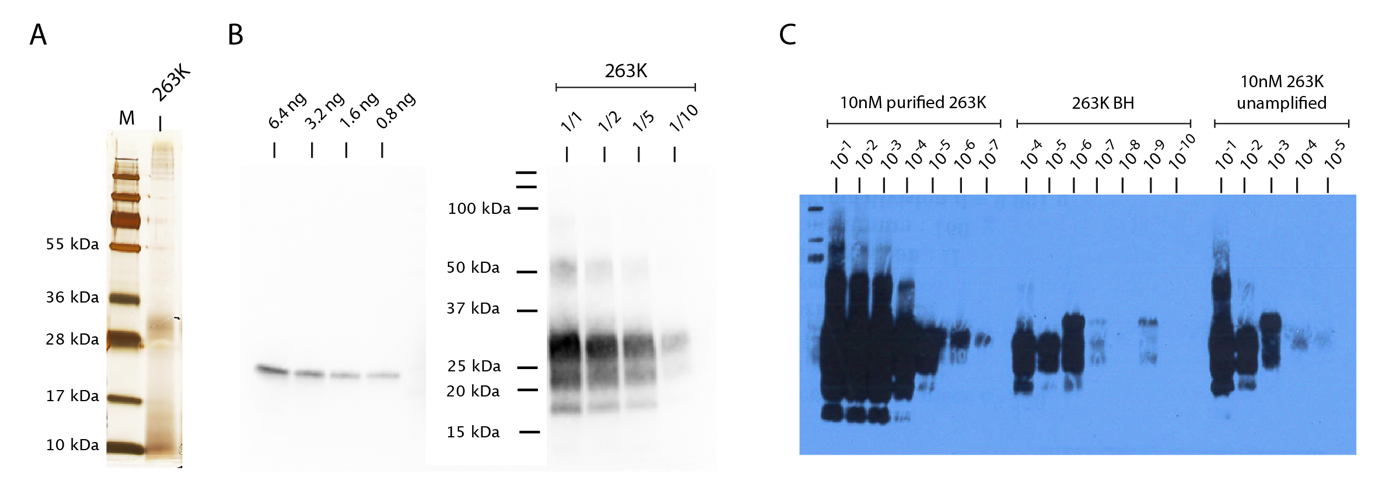
**

## S3 Appendix: PMCA templating activity of purified 263K PrP^Sc^ assemblies

263K PrP^Sc^ was purified as previously described by Baron and collegues ^1^, with some modifications. Briefly, 263K PrP^Sc^ was purified from 20% 263K-infected hamster brain homogenate. After the addition of one volume of CBS 2x (40 mM citrate pH 6.0, 274 mM NaCl), and 2 volumes of 1% (w/v) Brij-96 in CBS 1x, the solution was incubated for 30 min at 4°C. The sample was then adjusted to 26% OptiPrep (in 10 mM citrate pH 6.0, 137 mM NaCl) and split equally across 3 centrifuge tubes (13 mL). This fraction was overlaid with layers of 23% (12 ml) and 8% (10 ml) OptiPrep (in 10 mM citrate pH 6.0, 137 mM NaCl,) followed by 2 ml of 10 mM citrate pH 6.0, 137 mM NaCl). The gradients were centrifuged in a Beckman SW32 rotor at 18.500 rpm for 2 hr at 4°C. The 8%/23% OptiPrep interface was collected. A 1⁄2 volume of 5 M NaCl (in 0.15 M Tris pH 8.0) was added and the solution was incubated for 15 min at room temperature. The Optiprep concentration is adjusted to 26%. After splitting into 2 tubes (18 mL), the solution was overlaid with 15 ml of 23% OptiPrep in TBS (10 mM Tris pH8, 137 mM NaCl) and 4 ml of TBS. The gradients were centrifuged in an SW32 rotor at 18500 rpm for 2 hr at 20°C. The lipid band at the TBS/23% OptiPrep interface was carefully collected. After the addition of Sarkosyl (2% final concentration in 50 mM Tris pH 7.4), 25 U/mL of Benzonase in 50 mM Tris pH 7.4 was added and incubated during 30 min at 37°C under agitation. Next, 0.1 mg/mL of pronase was added and incubated for 30 min at 37°C under agitation. The supernatant was adjusted to contain a final concentration of 1.8 M NaCl. The solution was added to overlay a 1-volume sucrose solution (1 M sucrose, 0.1 M NaCl, 0.5% sulfobetaine 3-14, 10 mM Tris pH 7.4). After centrifugation at 30000 g for 2 hr at 16°C, the supernatant was carefully removed without disturbing the pellet. The pellet was washed by resuspension in 1/10th volume of 0.5% sulfobetaine (in PBS) followed by centrifugation at 30000 g for 2 hr at 16°C. The pellet was resuspended in water and precipitated by the addition of 3 volumes of cold acetone. The solution was incubated at -20°C overnight and centrifuged at 20000 g for 30 min. Finally, the pellet was resuspended in water and kept at 4°C. SDS-PAGE followed by silver-staining leads us to estimate the purity level of PrP^Sc^(**A**). 263K PrP^Sc^ concentration was estimated by using calibrated western blot with recombinant PrP (**B**). (**C**) The templating activity of the of 10nM equivalent to monomer of purified 263K PrP^Sc^ was estimated by PMCA using hamster PrP transgenic mouse brain as substrate. As shown in panel C, 10nM of purified PrP^Sc^ (10nM purified 263K) has a templating activity until the 10^-6^ dilution. The 263K infected brain homogenate (263K BH) and the 10nM 263K purified but unamplified (10nM 263K unamplified) are respectively the control for comparing the 10nM purified 263K seeding activity to 263K brain homogenate and the contribution of 10nM purified PrP^Sc^ to PrP res signal.
