# Supplementary material for: Reversible unfolding of infectious prion assemblies reveals the existence of an oligomeric elementary brick
Source: PLoS Pathog. 2017 Sep 7;13(9):e1006557. doi: 10.1371/journal.ppat.1006557 (PMC5589264; doi:10.1371/journal.ppat.1006557)
Supplement: S4 Appendix — (DOCX) [file ppat.1006557.s004.docx]

A


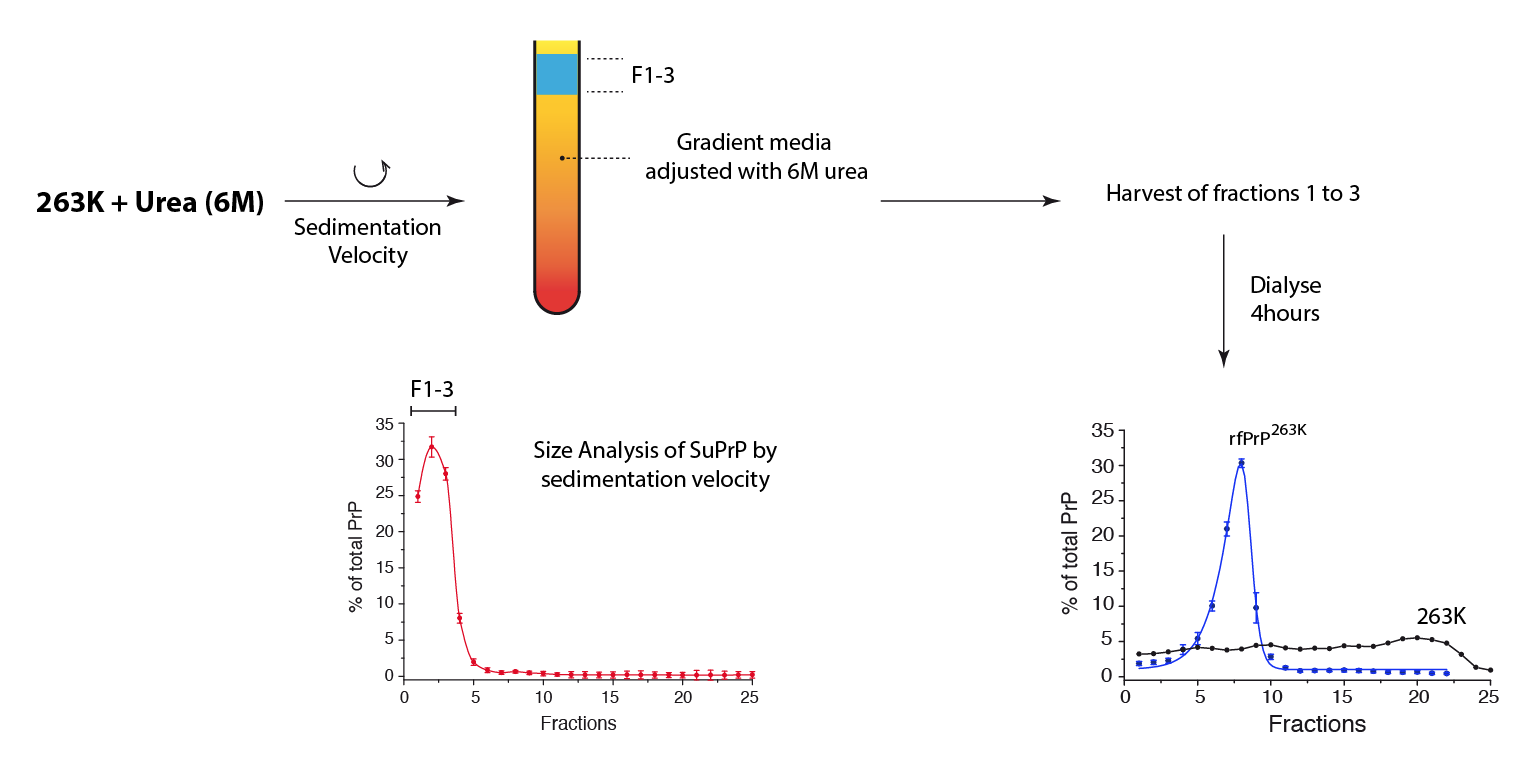


B


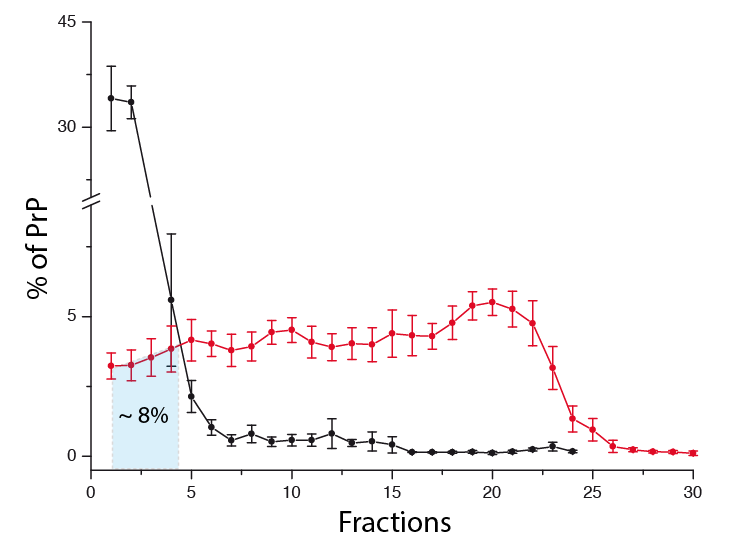


C


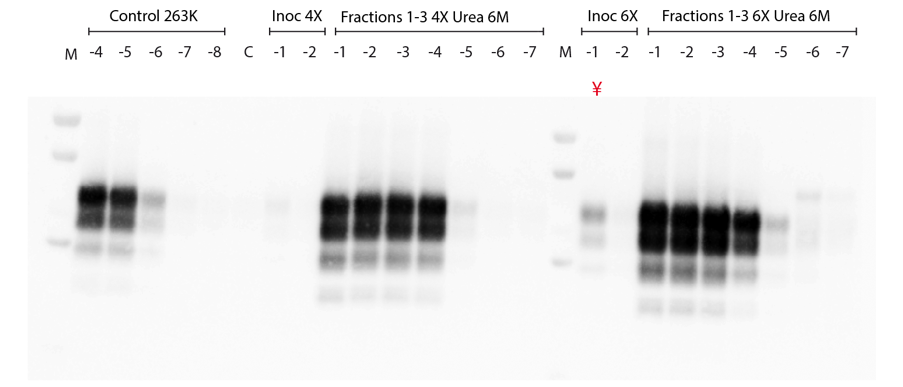


## S4 Appendix: The suPrP semi-purification procedure and the effect of suPrP concentration into rfPrP refolding.

(**A**) Procedure of suPrP263K semi-purification and its refolding after dialysis. (**B**) SV analysis of PrP content in healthy (black curves) versus 263K infected hamster brain (red curve). As reported on the figure, fractions corresponding to PrPC constitute less than 10% of the total PrP. (**C**) Acquisition of templating activity by concentrated suPrP after urea dilution. After SV containing 6M urea in the separation matrix the semi-purified suPrP fractions 1-3 corresponding to suPrP have been concentrated either 4 time (4X) or 6 time (6X) in volume using ultrafiltration with a 50KDa membrane cut-off. These concentrated fractions of suPrP in 6M urea have been used for PMCA amplification. C correspond to negative control of the PMCA, Inoc 4X and 6X correspond respectively to 4X and 6X without amplification at 10 and 100-fold diluted in PrP knockout brain homogenate (unamplified teste) in order to determine the contribution of initial inoculum signal to PMCA signal. Samples have been treated by PK and analysed by western blot as described in materials and methods. M indicates molecular weight.

A 10% 263K brain homogenate in 6M urea (or 8M for some specific experiments) was incubated at 37°C for a minimum of 1 hour, followed by a sedimentation velocity separation as previously described^2^. The media containing the iodixanol gradient was adjusted to 6M urea to prevent suPrP refolding during the sedimentation step. After fractions collection and western-blot analysis, fractions corresponding to fraction 1 to 3 were harvested and pooled. These fractions, containing mainly suPrP conformer were used for size exclusion chromatography (Figure 1E in the manuscript) or ultrafiltration concentration (Figure 4G in the manuscript).

It should be underlined that only in the case of 263K, the contamination by PrP^C^ can be neglected. Indeed, as shown in FigS4b, the red curve shows that a non-PK treated brain homogenate of 263K-infected hamster at end-stage of the disease seems to contain low amounts of PrP^C^, sedimenting in the upper fractions of the SV gradient. By comparing such distribution with that of uninfected hamster brain, one can estimate that a brain at terminal stage of disease contains less that 10% of PrP^C^ in the top fractions. This low PrP^C^ content is consistent with previous studies published by Safar and colleagues in Nature Medicine paper in 1998 (possibly due to PrP down-regulation, Mays et al, J.Virol 2015).

The two other prion strains used here T1^Ov-21K^ and T2^Ov-19K^) were isolated in tg338 mice, which over express 8-fold ovine PrP^C^. It was thus necessary to remove the PrP^C^ background by treating the samples with PK, as mentioned in material and methods section.

As stated in the manuscript, the process by which rfPrP is formed come from the condensation of suPrP. This process is a multi-molecular process, and thus is highly dependent on suPrP concentration. In PMCA experiments as well as for *in vivo* experiments, non-concentrated suPrP was diluted preventing its spontaneous condensation into rfPrP. By concentrating suPrP by 4- to 6-fold in 6M urea (by using ultrafiltration) before dilution into PMCA media is sufficient to initiate the formation of rfPrP, as shown by positive PMCA reactions (see S5 Appendix). It should be underlined that the simple 10-fold dilution of suPrP 6X in the PMCA media (inoc 6X, indicated by ¥ in S4C Appendix) leads suPrP to condensate into PK resistant assemblies
