# Supplementary material for: Reversible unfolding of infectious prion assemblies reveals the existence of an oligomeric elementary brick
Source: PLoS Pathog. 2017 Sep 7;13(9):e1006557. doi: 10.1371/journal.ppat.1006557 (PMC5589264; doi:10.1371/journal.ppat.1006557)
Supplement: S5 Appendix — (DOCX) [file ppat.1006557.s005.docx]

**
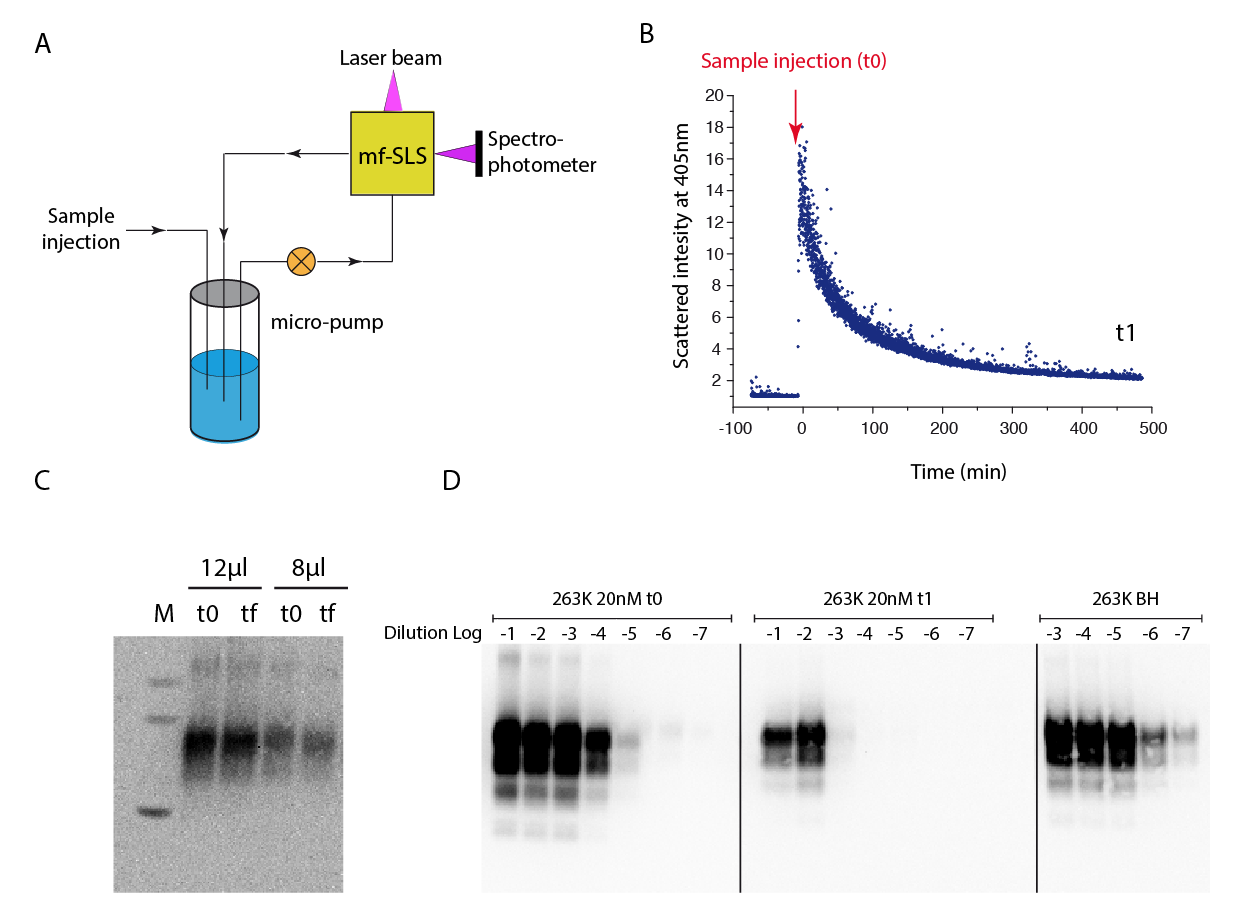
**

## S5 Appendix: Fast kinetic dilution and determination of size variation of PrP assemblies by light scattering intensity and templating activity assay by PMCA.

To study the size variation of PrP assemblies under diverse perturbation conditions, we built a homemade device using milli-fluidic system coupled to a homemade static light scattering device (SLS). The conception of this last leads to follow the size variation of protein aggregates in few molecules (i.e. low concentration). The high sensitivity of the SLS is mainly due to the use of laser beam with transvers mode TM00 with a waist of 2μm and a short wavelength (λ= 405nm, 450nm and 532nm).

It must be emphasised that due to the low concentration of prion assemblies, the use of conventional spectroscopy approaches such as Thioflavin-T probe is inefficient.

Our system of rapid dilution can be divided into two main compartment (**A**): The dilution compartment which include all the fluidic circuit and the detection system using the SLS. The fluidic circulation is performed by using piezo-micropump with a flow rate of 930μl/min. The total volume of the system is 430μl. The temperature of the system is precisely controlled using Peltier system and all experiments have been performed at 25°C.

As shown in (**B**) the purified 263K assemblies have been automatically injected in order to obtain 20nM equivalent to monomer at final concentration.

The variation of the molecular weight of a diluted solution containing heterogeneous objects is explored using Rayleigh relation:

$$\underset{C=0}{Lim} I\mathcal{=R.}\sum_{i=1}^{i} \tilde{C}_{i}{.M}_{i}.(1+ {cos}^{2}\theta)$$

where:

*I*: is the scattered intensity at angle θ;

*R*: Rayleigh constant by assuming that all objects have significantly identical dn/dc.

$\tilde{C}_{i}$: the mass-concentration of the i^th^ object and *Mi* its molecular weight.

*θ*: the detection angle here fixed at 88°

As θ is fixed we incorporate the term $(1+{cos}^{2}\theta)$ into Rayleigh constant. Then by assuming that the solution contains *i* objects with a molecular weight of *Mi* at the mass-concentration of *Ci* we can then transform the Rayleigh expression accordingly in order to estimate the weight average molecular weight (*<Mw>*) of object present in the solution. Indeed, we can define *<Mw>* as:

$$<Mw> =\sum_{i=1}^{i} \frac{\tilde{C}_{i}{.M}_{i}}{\tilde{C}_{i}}$$

$$<Mw> =\frac{\sum_{i=1}^{i} \tilde{C}_{i}{.M}_{i}}{\tilde{C}_{tot}}$$

By replacing $\sum_{i=1}^{i} \tilde{C}_{i}{.M}_{i}$ into Rayleigh relation we have:

$$\underset{C=0}{Lim} I\mathcal{=R.}\tilde{C}_{tot}.<Mw>$$

we can go further and express the light scattering intensity as a function of molar concentration and weight average of size of the object (i.e. number “*N*” of monomer repetition) (*<N>*):

$$\underset{C=0}{Lim} I\mathcal{=R.}C_{tot}.{<Mw>}^{2}$$

$$\underset{C=0}{Lim} I\mathcal{=R.}C.M^{2}.<N>$$

Where C is the concentration equivalent to monomer; M the molecular weight of the monomer and *<N>,* weight average of the size.

To determine the contribution of material adsorption on light scattering signal decrease, sample at t0 (immediately after dilution) and at final time (t1 = 8 hours) have been collected and analysed by western blot. As shown in (**C**), the amount of circulating PrP remains quasi constant as jugged when either 12μl or 8 μl has been deposit on SDS PAGE followed by western blot.

In (**D**) the templating activity of PrPSc assemblies has been tested by PMCA immediately after dilution (263K 20nM t0) and after almost 8 hours (263K 20nM t1). 263K BH corresponds to brain homogenate of hamster infected by 263K prion strain and used as positive control. The western blot has been performed as was described in the manuscript materials and methods using biotinylated Sha31 (Sha31b) antibody.
