# Supplementary material for: Reversible unfolding of infectious prion assemblies reveals the existence of an oligomeric elementary brick
Source: PLoS Pathog. 2017 Sep 7;13(9):e1006557. doi: 10.1371/journal.ppat.1006557 (PMC5589264; doi:10.1371/journal.ppat.1006557)
Supplement: S6 Appendix — (DOCX) [file ppat.1006557.s006.docx]

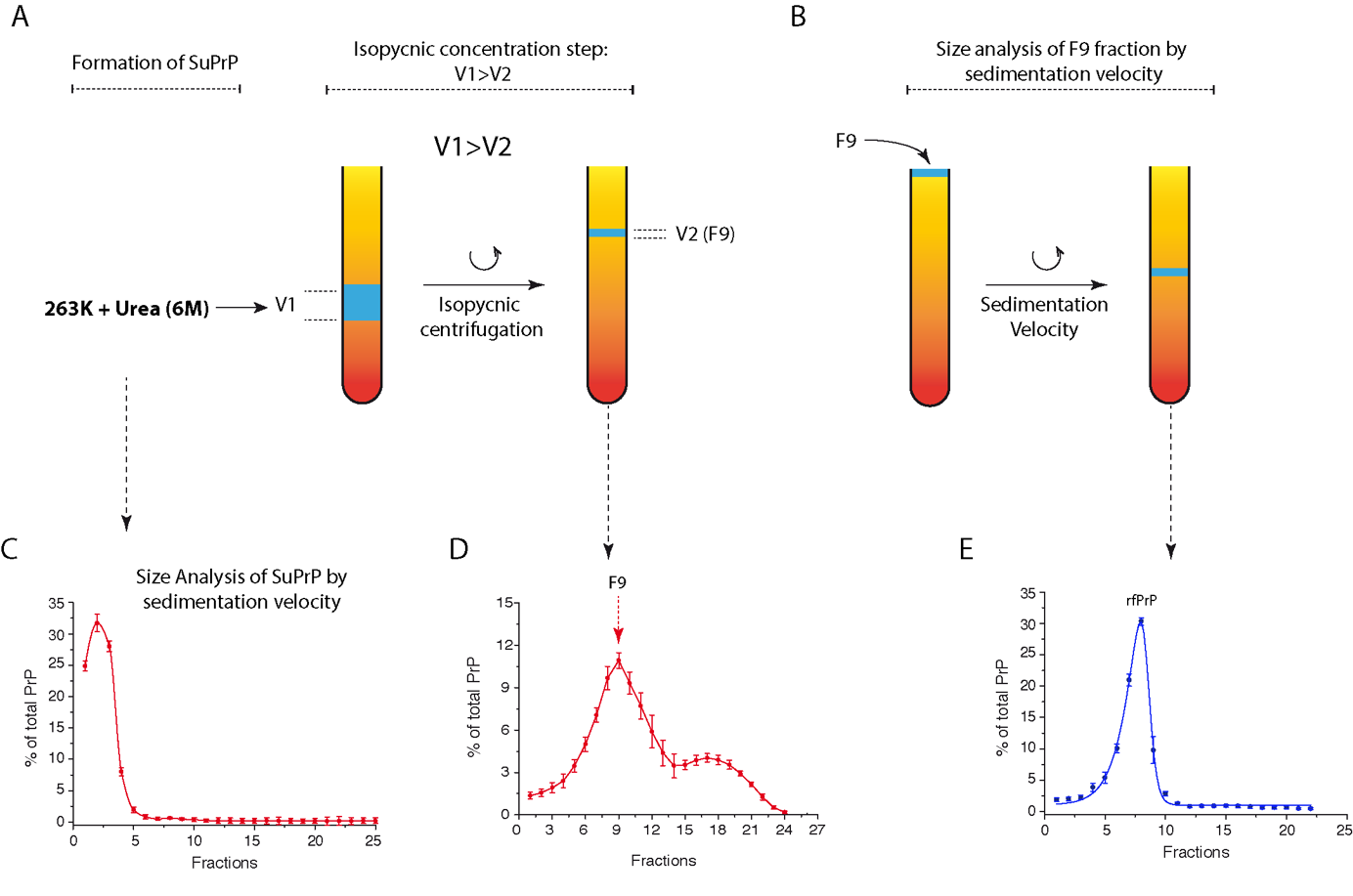


## S6 Appendix: Isopycnic concentration and refolding of SuPrP into rfPrP assemblies.

To directly observe the polymerization of SuPrP into higher assemblies, we first generate SuPrP by treating 263K assemblies with 6M urea and submitted the resulting mixture to isopycnic concentration as a method to concentrate and refold PrP. This last method also applicable with brain homogenate takes advantage of the different density properties of different compounds in order to i) dilute urea in the gradient, ii) and more importantly, increase locally SuPrP concentration (V1>V2) in a thin layer of the gradient (**A**). This last phenomenon triggers the condensation of SuPrP (**C**) into rfPrP. Indeed, the velocity sedimentogram analysis of fraction F9 of isopycnic sedimentation (**B**, **D**), revealed the apparition of a large assemblies corresponding to rfPrP (**E**).
