# Supplementary material for: Reversible unfolding of infectious prion assemblies reveals the existence of an oligomeric elementary brick
Source: PLoS Pathog. 2017 Sep 7;13(9):e1006557. doi: 10.1371/journal.ppat.1006557 (PMC5589264; doi:10.1371/journal.ppat.1006557)
Supplement: S7 Appendix — (PDF) [file ppat.1006557.s007.pdf]

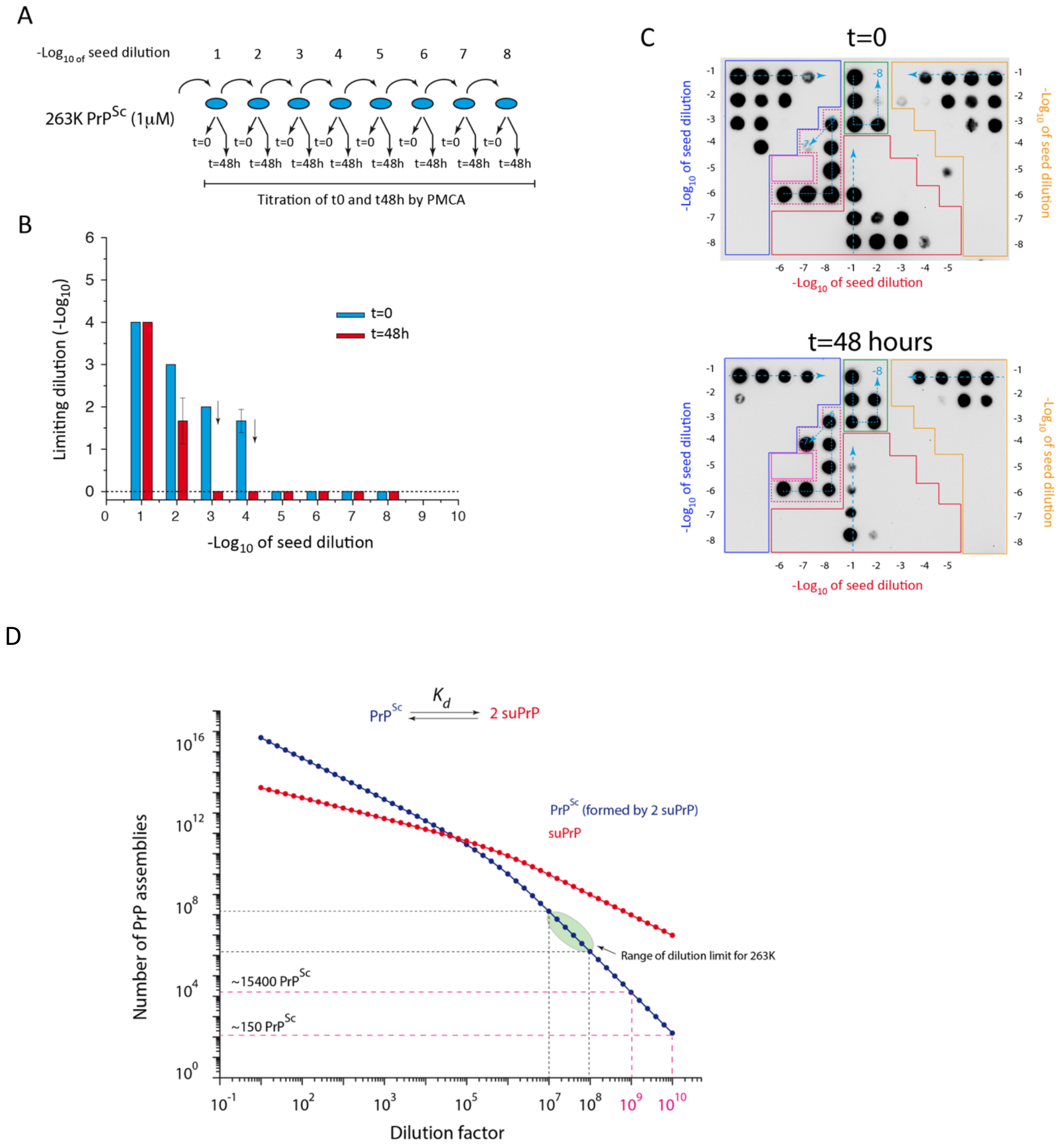

FigS7: The $PrP^{Sc} \rightleftarrows i.suPrP$ equilibrium displacement kinetic study by PMCA and the effect of high dilution.

(A) A solution containing 1µM purified 263K PrP^{Sc} was serially 10-fold diluted in a 20mM MOPS, pH 7.2 buffer. The dilutions were split in two batches of identical volume. The seeding activity of each dilution was titrated by PMCA immediately after dilution or after a 48h-incubation at 25°C. This step allows exploring the concentration- vs. time-dependency of PrP on $PrP^{Sc} \rightleftarrows i.suPrP$ equilibrium displacement.

(B) Titration of the seeding activity as a function of the initial PrP concentration at t0 (in blue) and t48h (in red). Arrows highlight the absence of detectable PrP^{res} signal after 48-hours incubation. The experiments have been done in triplicate with the same 263K PrP^{Sc} preparation.

(C) Dot blots analysis of PMCA PrP^{res}. The blue, red and orange frame correspond to each experiment constituting the triplicate. The limiting dilution raising to a PrP^{res}-positive PMCA for each concentration has been then determined by performing serial ten-fold dilution (the blue arrow in panel C indicates direction of dilution). As controls, purified 263K PrP^{Sc} (pink dot frame) and 10% 263K infected brain homogenate (green frame) were titrated for their PMCA activity.
(D) Variations of PrP^{Sc} and suPrP amounts with dilution. The starting material was fixed at 100nM of infectious PrP, which we assume corresponds to Ctot. The Kd was fixed to 10^{-9}. This value is in good accordance with the relaxation experience reported in Fig4 of the manuscript. As can be seen, a 10^{9}- and 10^{10}-fold dilution factor would correspond to respectively 15400 and 150 PrP^{Sc} (dimer of suPrP).

The experiments reported in Fig4 B showed that rapid dilution of purified PrP^{Sc} assemblies (from 1µM to 10nM) leads to a decrease in the mean molecular weight of PrP assemblies (i.e. a decrease in light scattering intensity). As shown in Fig4C, the size decrease is concerted with PMCA seeding activity (i.e. seeding activity at t1 compared to t0). These two observations suggest the existence of $PrP^{Sc} \rightleftarrows i.suPrP$ out of the chaotropic treatment context.
As the light scattering detection threshold did not allow exploring larger dilution factor of PrP^{Sc} assemblies (1nM, 0.1nM, …), we used the PMCA amplification technique. As shown in FigS7 panel A, purified 263K assemblies were serially ten-fold diluted in MOPS buffer pH7.2 and the volume of each dilution split in two batches. The first batch series was immediately analyzed by PMCA (our t0), whereas the second batch was analyzed by PMCA after a 48-hours incubation at 25°C.
As in Fig4C in the manuscript, one can observe a time-dependent disappearance of seeding activity, which supports the depolymerization of PrP^{sc} into suPrP by equilibrium displacement (B and C).
The existence of $PrP^{Sc} \rightleftarrows i.suPrP$ equilibrium has a consequence on limiting dilution estimation. By considering the equilibrium $PrP^{Sc} \rightleftarrows i.suPrP$ with a Kd that could be defined as: $K_d = \frac{suPrP^i}{PrP^{Sc}}$, with the total misfolded PrP concentration participating to this equilibrium: $C_{tot} = suPrP + i.PrP^{Sc}$., forlarge dilution, the size distribution will be centered around *i=2* according to size function partition distribution (i.e. larger assemblies will be disfavored). Thus by combining Kd and C_{tot} relations, we can easily deduce the relation that link the amount of suPrP to C_{tot}.

$$C_{tot} = 2PrP^{Sc} + suPrP$$

$$K_d = \frac{suPrP^2}{PrP^{Sc}}$$

$$2suPrP^2 + K_d.suPrP - K_d.C_{tot} = 0$$

$$suPrP = \mathcal{A}\frac{-K_d + \sqrt{{K_d}^2 + 8K_dC_{tot}}}{4}$$

$$PrP^{Sc} = \mathcal{A}\frac{C_{tot} - suPrP}{2}$$

A is the Avogadro number: 6.10^{23}

Now let's us assume that at the terminal stage of an animal the major part of PrP^C is converted in PrP^{Sc} and suPrP which is particularly true for 263K prion strain (Safar et el 1998). For hamster at the terminal stage, it would correspond roughly around 20 μg of PrP per gram of brain tissue (references [1, 2] and our estimation by immunoblot using Sha31 b and recPrP as calibration). It means C_{tot}≈8.10^{-10} mol/g brain. If we say in 5ml (i.e. 20% brain homogenate), C_{tot}=160nM of PrP. Let's consider a Kd value around nanomolar (Kd≈10^{-9}), which should be biochemically relevant considering the dilution range used in Fig 4B of the manuscript that leads to a depolymerization. Now using these roughly estimated parameters, we can plot PrP^{Sc} (corresponding to the condensation of two suPrP) and suPrP as function of dilution factor (D). As we can observe by taking a Kd value of 10^{-9} at a dilution factor of 10^9, we still have roughly 15400 molecules of PrP in the PrP^{Sc} conformation (here it corresponds to a dimer of suPrP). Even at the 10^{10} dilution, we have around 150 PrP^{Sc} assemblies. However it should be emphasize that this amount is strongly dependent on Kd and it cannot be excluded (and probably this is the case) that different prion strains present different Kd values.

1. Bolton DC, Rudelli RD, Currie JR, Bendheim PE. Copurification of Sp33-37 and scrapie agent from hamster brain prior to detectable histopathology and clinical disease. J Gen Virol. 1991;72 (Pt 12):2905-13. doi: 10.1099/0022-1317-72-12-2905. PubMed PMID: 1684986.

2. Chen B, Morales R, Barria MA, Soto C. Estimating prion concentration in fluids and tissues by quantitative PMCA. Nat Methods. 2010;7(7):519-20. doi: 10.1038/nmeth.1465. PubMed PMID: 20512142; PubMed Central PMCID: PMCPMC4049222.
